# Neuropathological changes in the nucleus basalis of Meynert in people with type 1 or type 2 diabetes mellitus

**DOI:** 10.1007/s00401-025-02942-y

**Published:** 2025-09-29

**Authors:** Wei Jiang, Martin J. Kalsbeek, Felipe Correa-da-Silva, Han Jiao, Andries Kalsbeek, Dick F. Swaab, Sarah E. Siegelaar, Chun-Xia Yi

**Affiliations:** 1 Department of Endocrinology and Metabolism, Location AMC, Amsterdam University Medical Center, University of Amsterdam, Meibergdreef 9, 1105 AZ Amsterdam, The Netherlands; 2 Amsterdam Gastroenterology Endocrinology Metabolism, Amsterdam University Medical Center, Meibergdreef 9, 1105 AZ Amsterdam, The Netherlands; 3 Endocrine Laboratory, Department of Laboratory Medicine, Amsterdam University Medical Center, Location AMC, Meibergdreef 9, 1105 AZ Amsterdam, The Netherlands; 4 Netherlands Institute for Neuroscience, Meibergdreef 47, 1105 BA Amsterdam, The Netherlands

**Keywords:** Acetylcholine, Alzheimer’s disease, Hypoglycemia, Hyperglycemia, Glymphatic system, Nucleus basalis of Meynert

## Abstract

People with type 1 or type 2 diabetes mellitus (T1DM or T2DM) often experience cognitive impairment. We profiled cells in the nucleus basalis of Meynert (NBM) in postmortem human brain tissue to investigate the neuropathological changes. Sixty-eight postmortem NBM samples were grouped as T1DM, T2DM, and controls without diabetes, with Braak stage 0–II or III–VI. T1DM subjects had only Braak stage 0–II and were thus compared only to controls with a similar Braak stage and not subjects with Braak stage III–VI. We analyzed neurons expressing choline acetyltransferase (ChAT), phosphorylated tau, amyloid-beta, glial cells, and vasculature with their respective markers. We found significantly lower neuronal expression of ChAT in T1DM individuals than in controls and T2DM individuals with Braak stage 0–II. Later-stage hyperphosphorylated tau levels were higher in T2DM compared to controls with Braak stage III–VI. Our results suggest that reduced acetylcholine production by NBM neurons may underlie the cognitive complaints of people with T1DM. In contrast, T2DM may exacerbate neuropathological changes associated with Alzheimer’s disease-like alterations.

**Supplementary Information:**

The online version contains supplementary material available at 10.1007/s00401-025-02942-y.

## Introduction

People with type 1 or type 2 diabetes mellitus (T1DM or T2DM) often experience cognitive impairment and are at an increased risk of developing Alzheimer’s disease (AD) [64, 79]. The etiology of T1DM and T2DM is different: T1DM is caused by an abnormal immune response that attacks and destroys insulin-producing beta cells in the pancreas, and it is often diagnosed at a young age [58]. In contrast, the main driving factors for T2DM are overweight/obesity, sedentary lifestyle, and consumption of unhealthy diets, characterized by insulin resistance, and typically T2DM develops later in life [80]. These differences are also associated with differences in cognitive impairment, as a previous study found that people with T1DM had more severe cognitive impairment than those with T2DM [36]. Yet, despite extensive cognitive assessments, neuroimaging, and epidemiological studies [36, 45, 64, 74, 80], the brain mechanisms at the cellular level underlying these cognitive impairments remain unclear, particularly those related to cognitive complaints in people with T1DM.

Acetylcholine (ACh)-producing neurons in the cholinergic nucleus basalis of Meynert (NBM) are crucial for cognition. Dysfunction of these neurons leads to cognitive impairment such as memory loss, learning difficulties, mood disorders, and attention deficit [12, 13, 22, 35, 38, 44, 72]. Previous studies have shown that cognitive decline is associated with neuronal atrophy and an increase in hyperphosphorylated tau (p-Tau) in the NBM [54, 81]. Surrounding these neurons, an essential supporting unit is formed by microglia, astrocytes, blood vessels, and the glymphatic system. These cells maintain brain homeostasis, ensure neuronal survival, and clear brain waste [20, 25, 27, 42, 65, 71, 76]. In particular, microglia-driven neuroinflammation is known to be pivotal in the pathogenesis of AD [37], whereas studies on the glymphatic system have produced conflicting findings in AD brains [55, 68].

Given these observations, we systematically profiled the neurons in the NBM using markers for choline acetyltransferase (ChAT, the key enzyme that catalyzes the biosynthesis of ACh) and Golgi matrix protein GA130 for neuronal activity [9, 12, 29]. We also examined three p-Tau markers (CP13, PHF1, AT8) associated with early- or late-stage of dementia [51], amyloid-beta (Aβ), microglia (using ionized calcium-binding adaptor molecule 1, Iba1), astrocytes (using glial fibrillary acidic protein, GFAP), vasculature (using alpha-smooth muscle actin, alpha-SMA, a major structural protein expressed by arteries and arterioles [63]), and the glymphatic system (using aquaporin 4, AQP4 [27, 42]). Our goal was to investigate whether the NBM was affected according to these parameters in T1DM or T2DM and to reveal the mechanisms linking diabetes to AD.

## Materials and methods

### Subject information

All brain material was obtained from the Netherlands Brain Bank. The donor or their next of kin gave informed consent for a brain autopsy and for the use of the brain material and medical records for research purposes [34]. Cases with severe neuropathologies unrelated to our study focus were excluded, unless mentioned otherwise in Table 1. In total, 68 postmortem human NBM samples from people with T1DM or T2DM and non-diabetic controls were studied (Tables 1 and 2). The average age of T1DM onset was 18 ± 3.5 years, and the average duration of T1DM was 42 ± 7.8 years (individual onset ages are listed in Table 1). There were no statistically significant differences in age at death between the groups, as assessed by non-parametric tests (*p* > 0.05) (Table 2). To ensure a fair comparison without the influence of neuropathological changes associated with AD or Parkinson’s disease, we grouped the subjects based on their Braak staging, which reflects the distribution of p-Tau throughout the brain rather than the intensity of p-Tau accumulation [6], and Lewy body distribution respectively [8].

**Table 1:**
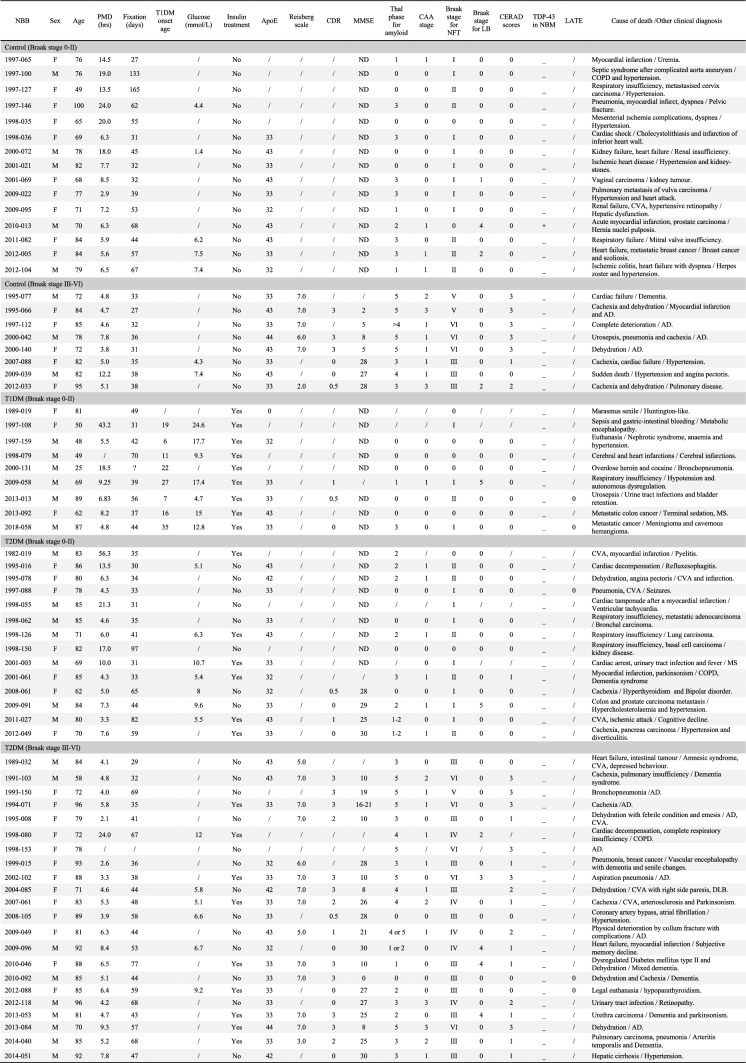
Clinico-pathological details of subjects

Subject 2012–118, 2014–051, 2018–058 also have Aging-related tau astrogliopathy (ARTAG neuropathology)*AD* Alzheimer’s disease, *ApoE* ε type of apolipoprotein E, *CAA* Cerebral Amyloid Angiopathy, *CDR* clinical dementia rating, *COPD* chronic obstructive pulmonary disease, *CVA* cardiovascular accident, *DLB* dementia with Lewy bodies, *LATE* Limbic-predominant age-related TDP-43 encephalopathy, *LB* Lewy bodies, *MMSE* Mini-Mental State Examination, MS Multiple sclerosis, *NBB* Netherlands Brain Bank, *ND* no dementia, *NFT* Neurofibrillary tangles, *PMD* postmortem delay, */* value unknown

**Table 2:** Subjects group characteristics

	Control *n* = 15 (Braak stage 0–II)	Control *n* = 8 (Braak stage III–VI)	T1DM *n* = 9 (Braak stage 0–II)	T2DM *n* = 14 (Braak stage 0–II)	T2DM *n* = 22 (Braak stage III–VI)	*p* value^a^	*p* value^b^
Age (years)	75.2 ± 11.24	81.25 ± 7.50	62.22 ± 21.34	78.87 ± 7.27	82.64 ± 9.57	0.12	0.78
Female (*N*, %)	10 (67%)	5 (63%)	3 (33%)	7 (50%)	13 (59%)	0.30	> 0.99
Male (*N*, %)	5 (33%)	3 (38%)	6 (67%)	7 (50%)	9 (41%)		
PMD (hrs)	11.06 ± 1.69	5.98 ± 0.98	13.75 ± 5.20	11.90 ± 13.83	6.11 ± 4.47	0.61	0.98
FT (days)	60.67 ± 37.39	40.88 ± 14.40	45.56 ± 10.97	46.43 ± 20.50	50.33 ± 13.80	0.53	0.08

Data are presented as mean ± SEM

*PMD* postmortem delay, *FT* fixation time

^a^Control vs. T1DM vs. T2DM in Braak stage 0–II

^b^Control vs. T2DM in Braak stage III–VI

Among all the 10 people with T1DM, Braak stages analysis using AT8 immunohistochemistry (AT8-ir) [6] showed that 9 of them had Braak stage 0–II. Therefore, we sub-grouped all non-diabetic, T1DM, and T2DM subjects into two categories: with Braak stage 0–II (associated with no cognitive decline) or Braak stage III–VI (associated with mild or severe cognitive decline) [6]. The non-diabetic control groups consisted of 23 subjects, of which 15 subjects with Braak stage 0–II and 8 with Braak stage III–VI. The T2DM group was composed of 14 subjects with Braak stage 0–II and 22 subjects with Braak stage III–VI (Tables 1 and 2). In addition, all groups were matched for sex, age, fixation time, postmortem delay (PMD), and pH in cerebrospinal fluid (CSF) (a measure for agonal state) to prevent the potential impact of confounding factors (Table 2).

A comprehensive overview of each individual case is provided in Table 1, including sex, age at death, postmortem delay (PMD), fixation duration (days), age of onset of T1DM, ApoE sub-genotype, Reisberg Scale [56], Clinical Dementia Rating (CDR) [47], Mini-Mental State Examination (MMSE) score [14], glucose levels (mmol/L), insulin treatment history, clinical diagnosis, and cause of death. The neuropathological assessment was performed by board-certified neuropathologists for each subject according to the National Institute on Aging-Alzheimer’s Association guidelines [46], which include the Braak stage for neurofibrillary tangles [7], amyloid deposition [70], and CERAD (Consortium to Establish a Registry for Alzheimer’s Disease) score [46]. Additionally, age-related neuropathological changes—such as Lewy body pathology [8], limbic-predominant age-related TDP43 encephalopathy (LATE) [50], and cerebral amyloid angiopathy (CAA) [69]—are also documented. These clinical and pathological measures reflect both cognitive status and disease progression: for example, the ApoE ε4 allele is a genetic risk factor for Alzheimer’s disease; higher Braak stage and Thal phases indicate more severe tau and amyloid pathology, respectively; the Reisberg Scale and CDR assess the severity of functional and cognitive impairment, while MMSE evaluates global cognitive function, with lower CDR and higher MMSE scores indicating better cognitive performance.

### Histology and morphometry of the NBM

**Fig. 1:**
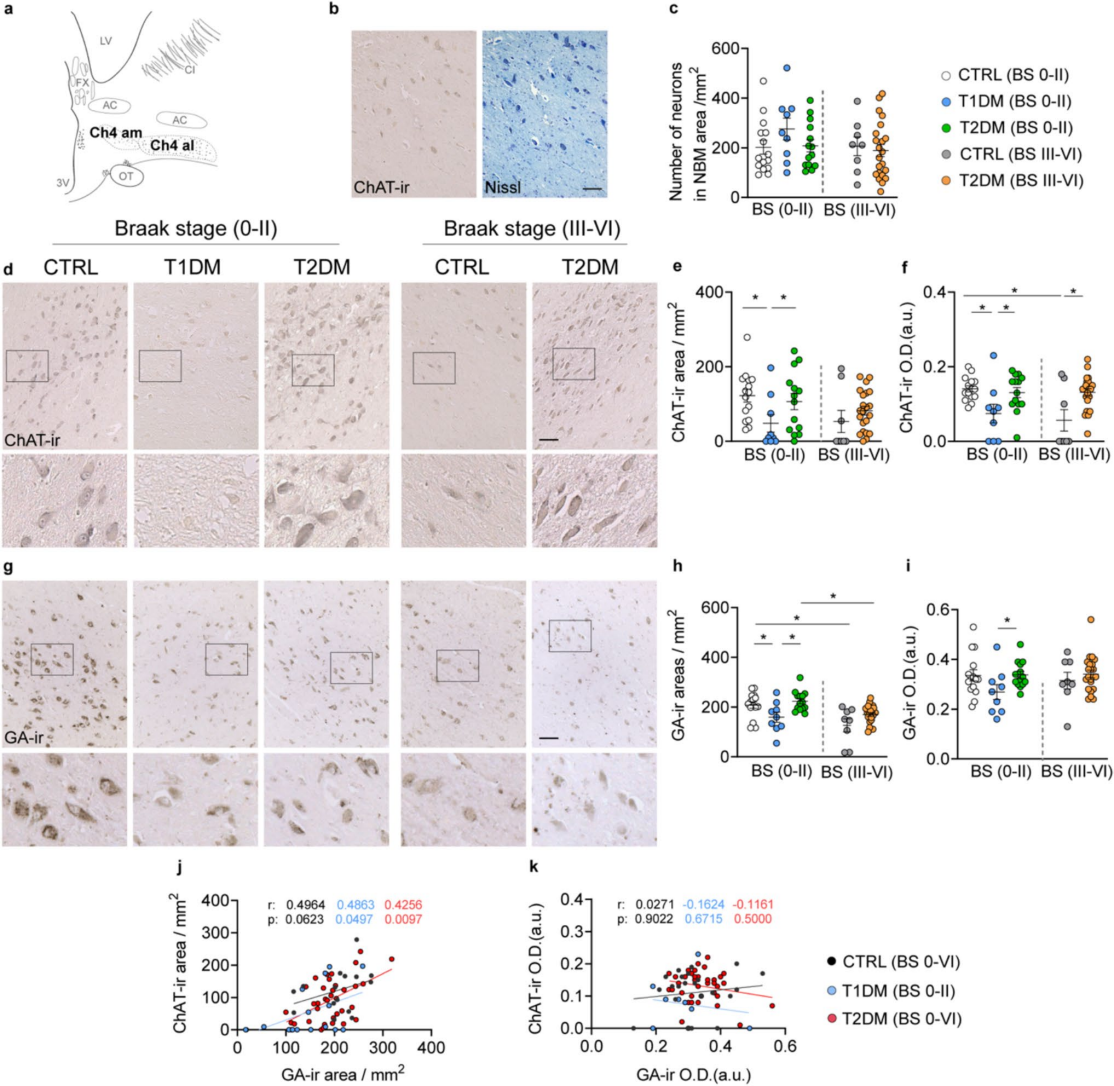
Choline acetyltransferase immunoreactive (ChAT-ir) and Golgi matrix protein GA130 immunoreactive (GA-ir) neurons are reduced in the NBM of T1DM, but not T2DM, subjects**. a** Schematic representation of the medial part (Ch4-am) and the lateral part (Ch4-al) of the nucleus basalis of Meynert (NBM) area used for cell profiling, created based on the Handbook of Clinical Neurology Chapter 2 (HCN #79) [67]. 3 V, third ventricle; AC, anterior commissure; CI, capsula interna; FX, fornix; LV, lateral ventricle; ON, optical nerve. **b** Representative images of Nissl-stained cells and ChAT-ir cells in the same T1DM subject; **c** Neuron density visualized by Nissl staining in the NBM of control, T1DM, and T2DM subjects over different Braak stages is comparable. **d** Representative images of ChAT-ir in the NBM of the control, T1DM, and T2DM subjects; high-magnification views of the boxed areas are shown in the lower panels. **e, f** Comparisons of ChAT-ir area and optical density (in arbitrary units, O.D. (a.u.)) of ChAT-ir neurons between controls, T1DM, and T2DM Braak stage 0–II or III–VI. **g** Representative images of GA-ir in the NBM of the controls, T1DM, and T2DM subjects; high-magnification views of the boxed areas are shown in the lower panels. **h, i** Comparisons of GA-ir area and O.D. of GA-ir neurons between controls, T1DM, and T2DM with BS 0–II or BS III–VI. **j, k** Plots of GA-ir area and O.D. with ChAT-ir O.D. Scale bar: 100 µm in d, g. Data are represented as mean ± SEM. * p < 0.05

After autopsy, the isolated brain tissues containing the hypothalamus and NBM were immediately immersed in 10% formalin and fixed at room temperature for 1–2 months. The tissues were then ethanol-dehydrated, toluene-cleared, and paraffin-embedded. Serial 6 μm coronal sections were made along the rostro-caudal axis, including the NBM area. The anatomical orientation and rostro-caudal range of the NBM were determined by Nissl staining (Supplementary information) and confirmed by ChAT immunoreactivity (ChAT-ir). Since the NBM consists of a large cell population, we performed our studies in a standardized region of the NBM, as described in previous studies [12, 43, 81], specifically the anteromedial (Ch4am) and anterolateral (Ch4al) subregions of the NBM at the level of the fornix and/or the anterior commissure [67] (Fig. 1a). All immunohistochemical or immunofluorescent staining was performed on consecutive sections at this level. For ChAT-ir, three sections per brain were selected: the level with peak neuron density in the NBM and sections 600 μm (100 sections) anterior and posterior to it. For other staining, one section per subject at the level of peak neuron density in the NBM was used.

### Immunohistochemistry and immunofluorescence

For antibodies of ChAT, GA130, CP13, PHF1, Iba1, GFAP, Aβ, TDP-43, and alpha-SMA, we performed epitope retrieval to enhance antigen exposure (Supplementary information), whereas AT8 and AQP4 antibody did not require epitope retrieval. After these procedures, sections were incubated with the primary antibody diluted in SUMI buffer (0.25% gelatine, 0.5% Triton X-100 in TBS (pH 7.6), except for the alpha-SMA and GFAP antibodies, which were diluted in SUMI containing 10% donkey serum. All primary antibodies—used either for single staining or for co-staining of ChAT with CP13 or PHF1—were incubated overnight at 4 °C. For immunohistochemistry, after overnight incubation, sections were incubated with the corresponding biotinylated secondary antibody, followed by avidin–biotin complex (ABC). The chromogenic reaction was then performed with 3,3′-Diaminobenzidine with ammonium nickel sulfate to improve contrast, except for sections stained for p-tau and Aβ, which were developed with DAB alone due to the overly intense staining resulting from DAB-nickel (Supplementary information). All reactions were stopped in distilled water, ethanol dehydrated, xylene cleared, and cover-slipped with Entellan mounting medium.

For immunofluorescence single staining of alpha-SMA and GFAP, biotinylated secondary antibody against each of these primary antibodies was incubated, followed by incubation with a fluorophore-conjugated Streptavidin. For immunofluorescence co-staining of ChAT with CP13 or PHF1, primary antibodies (ChAT with CP13 or ChAT with PHF1) were incubated overnight, biotinylated secondary antibody against ChAT was incubated, followed by incubation with a fluorophore-conjugated secondary antibody against the CP13 or PHF1 together with a fluorophore-conjugated Streptavidin. The list of primary antibodies and information on their specificity is shown in Supplementary Table 1.

### Imaging acquisition and quantitative analysis

Immunohistochemistry images were acquired using a Zeiss Axio Scanner and analyzed with QuPath software. The NBM was manually delineated at 20 × magnification, total Nissl neuron density was calculated by dividing the number of Nissl-stained cells by the total NBM area. For each immunostaining, the number of immunoreactive (ir) areas—corresponding to neurons—was quantified within the NBM as defined based on the Nissl-stained boundaries. The “Intensity Features” tool in QuPath was used to quantify the average number of -ir areas (i.e., neurons), which was normalized to background levels. Cell density was expressed as the number of -ir areas per mm^2^ (ir area/mm^2^). Neurons are also quantified with the optical density (O.D.), a threshold for positive immunoreactivity was set at two times the optical density of the background. Optical density was used to quantify all data, as results were consistent when using integrated optical density (IOD = O.D. × % area) as an endpoint.

For AQP4 immunoreactivity, images were captured using Image Pro version 6.3 (Media Cybernetics), and the relative positive area was quantified with the “Analyze Particles” tool in ImageJ. Iba1-positive microglial soma was quantified as “particles” with sizes ranging from 20 µm^2^ to 100 µm^2^ in ImageJ, based on criteria defined in a previous study [31]. Images from immunofluorescence staining of α-SMA and GFAP were acquired using a Zeiss Axio Scanner and was measured using the “Pixel Classification” tool in QuPath. Pictures of immunofluorescence co-staining of ChAT with CP13 or PHF1 were acquired using a Leica SP8 confocal microscope. Co-localization of ChAT with CP13 or PHF1 was manually assessed by counting single- and double-labeled cells.

### Statistical analysis

Following assessment of normality using the D’Agostino and Pearson test, pairwise comparisons within the Braak stage 0–II group or the Braak stage III–VI group were conducted using Mann–Whitney *U* tests. Comparisons between control subjects with Braak stage 0–II or Braak stage III–VI and T2DM subjects with Braak stage III–VI were also conducted using Mann–Whitney U tests. To identify differences among the three groups, the Kruskal–Wallis test was applied due to the non-normal distribution of the data. Post hoc *p* values were corrected for multiple comparisons using the Benjamini–Krieger–Yekutieli method. Confounder analyses were evaluated using Spearman’s rank correlation coefficient. A *p* value ≤ 0.05 was considered statistically significant. To determine whether the strength of observed correlations differed between cohorts, Fisher’s z-test was applied to the correlation coefficients of both positive and negative correlations data. All statistical analyses were performed using GraphPad Prism 9.5.1. and RStudio 4.4.2.

### Confounder analyses

Considering the potential impact of biological and pathological variabilities in the human subjects before and during death, including age, PMD affecting tissue degradation, pH of CSF, blood glucose levels during lifetime, tissue fixation time, and Braak stage, we ensured maximum matching of these parameters across all groups (Table 2). Furthermore, given the overall sample number is modest, we have conducted simple linear regression analyses between these factors and the major study outcomes (Figs. S1–S15).

## Results

### Diverse alterations of cholinergic neurons in the NBM of individuals with T1DM or T2DM.

We first quantified the density of NBM neurons per delineated NBM area using Nissl staining, soma size between 30 µm^2^ and 300 µm^2^ was defined as a neuron. Neuron density of control, T1DM, and T2DM subjects across different Braak stages was comparable (Fig. 1b, c). Moreover, in confounder analysis, we found no correlations between neuron density and age, PMD, pH of CSF, tissue fixation time, blood glucose level, or Braak stage (Fig. S1). However, in the NBM of T1DM subjects, we found a significantly lower ChAT immunoreactive (ChAT-ir) area and optical density (O.D.) of ChAT-ir. This was not observed in controls and T2DM subjects with Braak stage 0–II (Fig. 1d–f). In controls with Braak stage III–VI, we observed a significantly lower optical density of ChAT compared to controls with Braak stage 0–II. Interestingly, in T2DM subjects with Braak stage III–VI, the ChAT-ir area was not smaller than in controls and T2DM subjects with Braak stage 0–II. Moreover, the ChAT-ir optical density was significantly higher in T2DM subjects with Braak stage III–VI than in controls of the same Braak stage group (Fig. 1d–f).

ChAT-ir neuronal morphology in the NBM varied between groups. In controls and individuals with T2DM at Braak stage 0–II, magnocellular ChAT-ir neurons typically exhibited one, or occasionally two, proximal dendrites. In contrast, ChAT-ir intensity was markedly reduced in individuals with T1DM and in controls at Braak stage III–VI, with proximal dendrites and fibers appearing barely visible. Notably, in T2DM subjects at Braak stage III–VI, ChAT-ir morphology appeared largely preserved. These findings suggest that cholinergic neuronal integrity is selectively impaired in T1DM, while changes observed in T2DM may reflect compensatory or distinct pathophysiological mechanisms.

Furthermore, since CDR and MMSE scores were available for some control and T2DM individuals, we examined whether ChAT-ir neurons in the NBM correlated with these cognitive measures. In control subjects, we observed a significant negative correlation between CDR and ChAT-ir area, and a significant negative correlation between CDR and ChAT-ir optical density in both control and T2DM subjects. For MMSE, there was a significant positive correlation between MMSE scores and ChAT-ir optical density in T2DM subjects, and a trend toward a positive correlation between MMSE and both ChAT-ir area and optical density in control subjects (Fig. S3). These findings support the notion that cholinergic dysfunction in the NBM is associated with cognitive decline.

As no clear difference was found in cell density in the NBM by Nissl histology, we determined whether the fewer ChAT-ir in the NBM of T1DM subjects was due to less neuronal metabolic activity. Therefore, we analyzed the metabolic activity of the NBM using GA130, which recognizes the Golgi matrix and has been validated as an indicator of neuronal activity in the NBM [12, 59]. We found that the GA-ir area and optical density were indeed lower in the NBM of T1DM subjects compared to controls and T2DM subjects (Fig. 1g–i). Moreover, consistent with previous findings [12, 59], the GA-ir area in controls and T2DM subjects with Braak stage III–VI were both significantly lower than those with Braak stage 0–II (Figs. 1g–i).

Morphologically, GA-ir in controls with Braak stage 0–II was prominent in the perinuclear cytoplasm of large, well-defined neurons, showing compact, granular patterns consistent with intact Golgi structures. In contrast, T1DM subjects exhibited shrunken and faint GA-ir signals (Fig. 1g), suggestive of compromised Golgi function. Importantly, we observed a significant positive correlation between GA-ir and ChAT-ir areas in both T1DM and T2DM subjects, with a similar trend present in controls (Fig. 1j, analyses of optical density are presented in Fig. 1k). These findings suggest that reduced metabolic activity in NBM neurons may underlie the cholinergic dysfunction observed in individuals with T1DM.

**Fig. 2:**
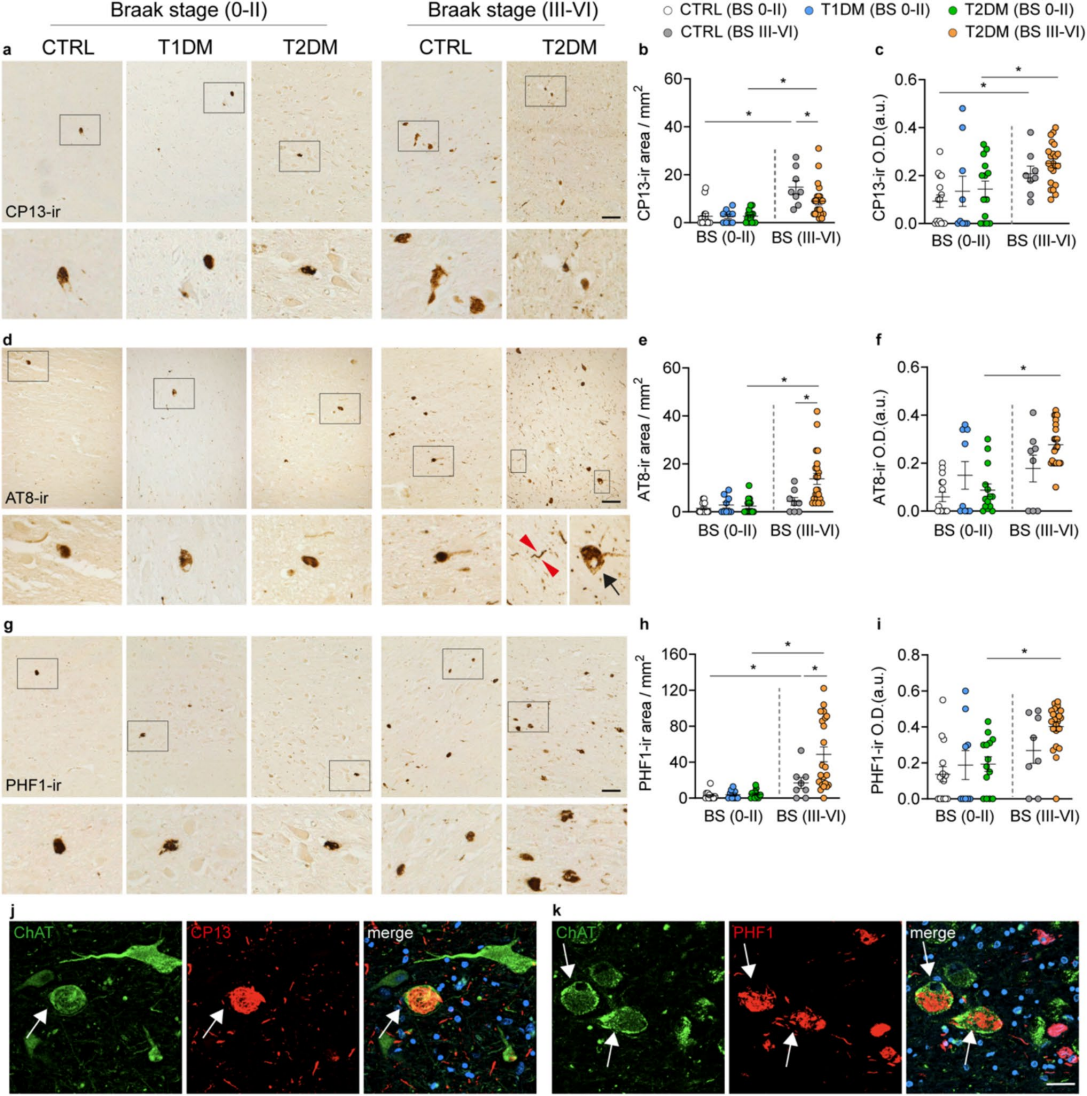
Phosphorylated tau (p-Tau) immunoreactivity is unchanged in the NBM of T1DM subjects with Braak stage 0–II, but increased in T2DM subjects with Braak stage III–VI. **a** Representative images of CP13-immunoreactive (CP13-ir) neurons in the NBM of control, T1DM, and T2DM subjects; high-magnification views of the boxed areas are shown in the lower panels. **b, c** Comparisons of the CP13-ir area and CP13-ir optical density (in arbitrary units, O.D. (a.u)) between controls, T1DM, and T2DM with Braak stage (BS) 0–II or III–VI. **d** Representative images of AT8-ir neurons in the NBM of control, T1DM, and T2DM subjects; high-magnification views of the boxed areas are shown in the lower panels. In the T2DM subject, typical AT8-ir neurites and neuropil threads (indicated by red arrows) and a globose structure (indicated by a black arrow) are highlighted in the upper panel and enlarged in the lower panels. **e, f** Comparisons of AT8-ir area and AT8-ir O.D. neurons between control, T1DM, and T2DM with BS 0–II or BS III–VI. **g** Representative images of PHF1-ir neurons in the NBM of the control, T1DM, and T2DM subjects; high-magnification views of the boxed areas are shown in the lower panels of **g**. **h, i** Comparisons of PHF1-ir area and PHF1-ir O.D. between control, T1DM, and T2DM in BS 0–II or BS III–VI. **j, k** Representative images demonstrating co-localization of ChAT-ir with CP13-ir (**j**) or PHF1-ir (**k**) in the NBM of a T2DM subject with Braak stage III–VI. Cell nuclei were stained with DAPI (blue). Approximately 65% of ChAT-ir neurons co-express CP13, and 60% co-express PHF1. Arrows indicate cells with co-localization. The p-Tau tangles are visible in ChAT-ir panels in both **j** and **k**. Scale bar: 100 µm in **a**, **d,** and **g**, 30 µm in **k**. Data are represented as mean ± SEM. *p < 0.05

With respect to the major confounders (Figs. S2 and S4), we found a negative correlation between ChAT-ir areas with Braak stage in control subjects (Fig. S2f), and a negative correlation between the GA-ir areas and Braak stage in control and T2DM subjects (Fig. S4f), indicating the NBM neuronal activity declines along the course of cognitive decline.

### The impact of T1DM or T2DM on P-Tau and Aβ in the NBM

**Fig. 3:**
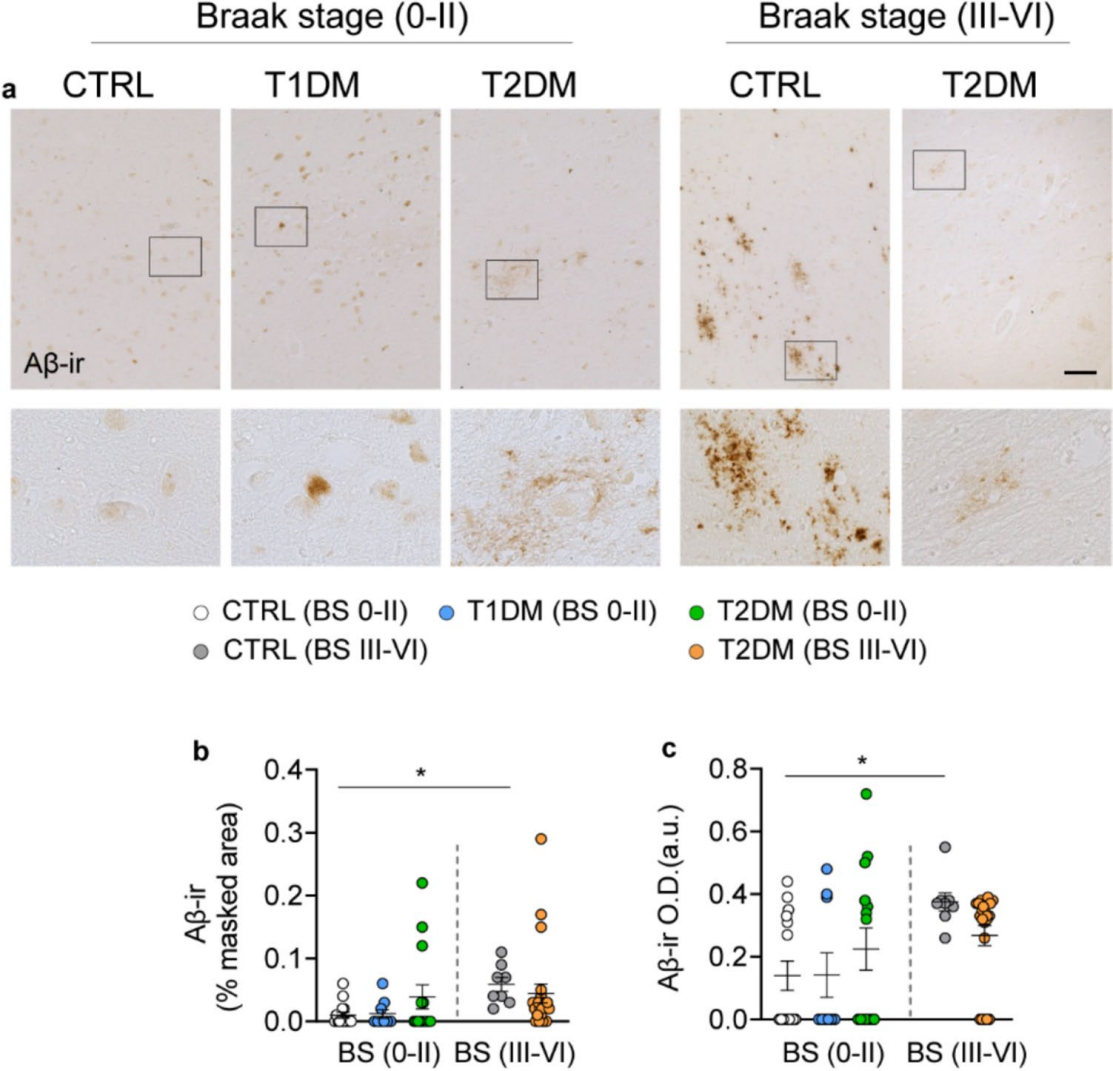
Amyloid-β immunoreactivity (Aβ-ir) is not altered in the NBM of T1DM or T2DM subjects with Braak stage 0–II, but is increased in control subjects with Braak stage III–VI. **a** Representative image of Aβ-ir plaques in the NBM of control, T1DM, and T2DM subjects with detectable Aβ-ir (only cases with visible Aβ-ir signals are shown, as most individuals with Braak stage 0–II exhibited minimal immunoreactivity), high-magnification views of the boxed areas are shown in the lower panels. **b, c** Comparisons of the Aβ-ir masked area (%) and Aβ-ir optical density (in arbitrary units, O.D. (a.u)) between controls, T1DM, and T2DM with Braak stage (BS) 0–II or III–VI. Scale bar: 100 µm in a. Data are represented as mean ± SEM. * *p* < 0.05

All T1DM subjects included in our analysis had Braak stage 0–II, as determined by routine pathological assessment using AT8-ir at the Netherlands Brain Bank. To assess whether these brains exhibited other early- or late-stage p-Tau markers, we examined two additional phosphorylated tau proteins: CP13, associated with early (pre-tangle) AD pathology, and PHF1, which appears at later stages of cognitive decline of AD [51]. Among subjects with Braak stage 0–II, there were no significant differences in AT8-ir, CP13-ir, or PHF1-ir areas between control, T1DM, and T2DM groups (Fig. 2). As expected, CP13 and PHF1 levels were significantly higher in control subjects with Braak stage III–VI compared to those with Braak stage 0–II, confirming the staging sensitivity of these markers. Notably, in T2DM subjects with Braak stage III–VI, the CP13-ir area was significantly lower than in stage-matched controls, while the AT8- and PHF1-ir areas were significantly higher (Figs. 2a–i), indicating a shift toward more advanced tau pathology in T2DM. Aβ immunostaining was also performed across all subjects. In general, most of the individuals with Braak stage 0–II showed minimal Aβ-ir (Figs. 3a–c, only cases with visible Aβ-ir signals are shown). As expected, in controls, Aβ-ir was significantly higher in those with Braak stage III–VI compared to Braak stage 0–II. However, in both T1DM and T2DM subjects with Braak stage 0–II, Aβ-ir levels did not differ significantly compared to the controls (Figs. 3a–c).

Morphologically, p-Tau in subjects with Braak stage III–VI displayed a globose shape and dense neuropil threads (Fig. 2d, AT8-ir). In contrast, p-Tau staining in controls and T1DM subjects with Braak stage 0–II was rare and typically presented as round or ovoid cytoplasmic inclusions, with little to no dendritic involvement and no neuropil threads (Figs. 2a, d, g). The observed increase in AT8-positive neuropil threads in advanced Braak stages likely reflects abnormal tau accumulation within neuronal processes. Neuropil threads are known to represent phosphorylated tau aggregates in axons and dendrites, and their presence suggests cytoskeletal abnormalities and impaired axonal transport—hallmarks of axonal dysfunction. These findings indicate that tau pathology in the NBM is not restricted to neuronal soma but also involves axonal compartments, supporting the presence of progressive neurodegenerative changes.

Moreover, in the NBM of T2DM subjects—where both ChAT and p-Tau were relatively abundant—not all ChAT-ir neurons expressed p-Tau. Specifically, approximately 65% of ChAT-ir neurons were positive for CP13, and 60% for PHF1 (Fig. 2j, k). Aβ-ir plaques in NBM in T2DM subjects with Braak 0–II were sparse and diffuse. In controls with Braak III–VI, dense-core plaques were observed in NBM, whereas in T2DM subjects with Braak III–VI, Aβ-ir appeared predominantly as diffuse or granular deposits, with fewer well-formed plaques (Fig. 3a). These findings suggest T1DM does not appear to accelerate AD-related tau or Aβ pathology in the NBM. In contrast, T2DM is associated with a shift toward more advanced tau pathology in the NBM, suggesting a potential role in exacerbating neurodegenerative processes.

Regarding the impact of major confounders on p-Tau expression (Figs. S5–S7), all three p-Tau markers showed a positive correlation between the immunoreactive areas or the optic density and the Braak stage in both control and T2DM subjects, indicating that p-Tau levels increase as cognitive decline progresses (Figs. S5f, l, S6f, l, and S7f, l). Additionally, CP13-ir and PHF1-ir areas were positively associated with age in controls (Fig. S5a; Fig. S7a). Furthermore, a negative correlation was observed between ChAT-ir and CP13-ir areas in the control group (Fig. S5m, n). None of the confounders showed a correlation with p-Tau expression in the NBM of T1DM subjects. Due to the minimal Aβ-ir observed in most individuals with Braak stage 0–II, confounder analysis was not performed for Aβ-ir.

### Lower microglial activity in the NBM of T1DM individuals

**Fig. 4:**
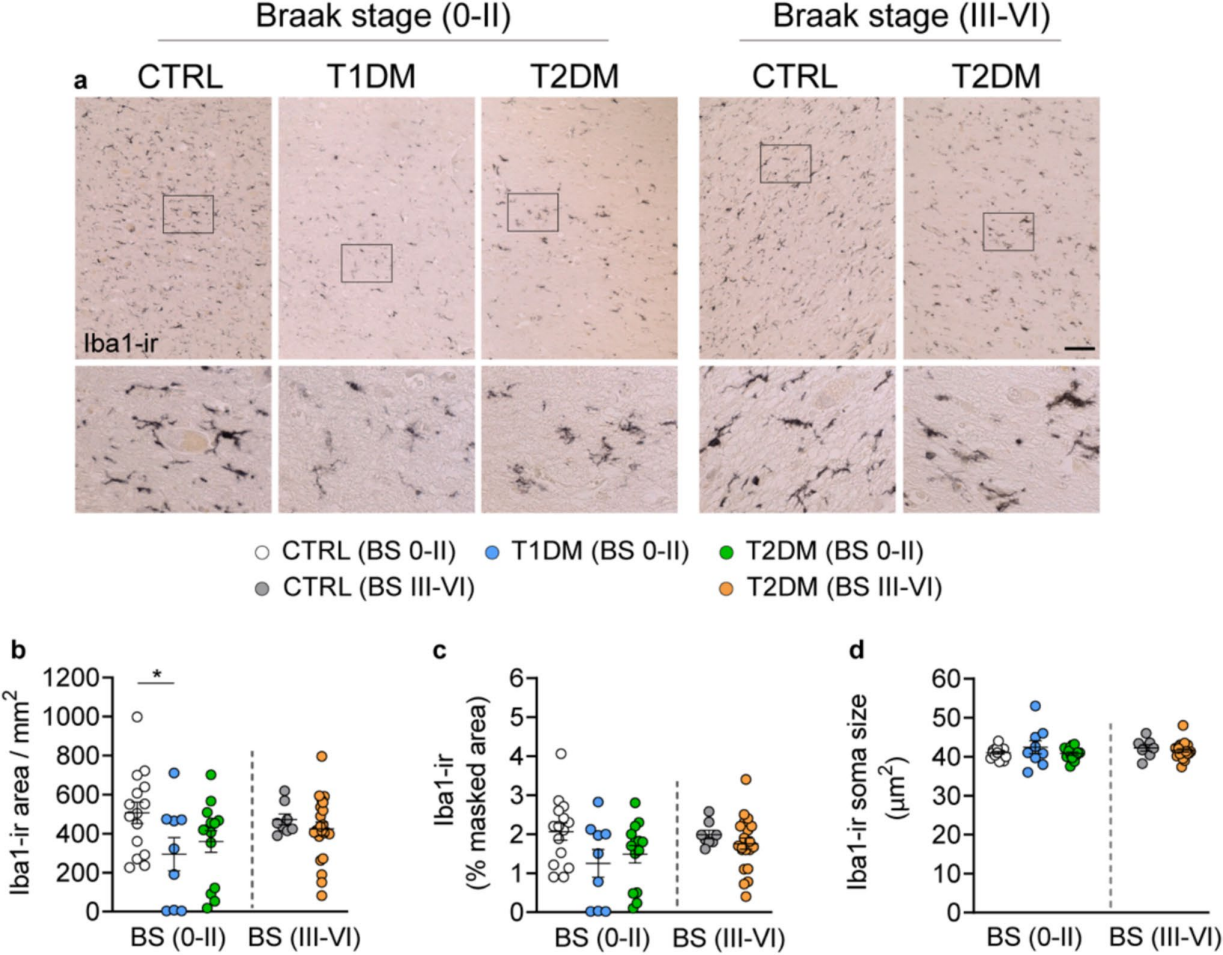
Reduced ionized calcium-binding adaptor molecule 1 (Iba1)-immunoreactive microglia in the NBM of T1DM subjects. **a** Representative images of Iba1-ir in the NBM of control, T1DM, and T2DM subjects; high-magnification views of the boxed areas are shown in the lower panels. **b, c, d** Comparisons of the soma density, masked area (%) and the soma size of Iba1-ir between control, T1DM, and T2DM with Braak stage 0–II or III–VI. Scale bar: 100 µm. Data are represented as mean ± SEM.* *p* < 0.05

Microglia-driven neuroinflammation is recognized as pivotal in the pathogenesis of AD [37]. However, among all the microglial parameters, *i.e.,* the soma density, masked area (%), and the soma size of Iba1-ir, we only observed a decrease in Iba1-ir microglial soma density in the NBM of T1DM subjects with Braak stage 0–II (Figs. 4a–d). No significant changes were observed in microglial cell density, soma size, or total Iba1-ir area in controls and T2DM subjects with Braak stage III–VI. Additionally, during confounder analysis (Figs. S8 and S9), we found a negative correlation between Iba1-ir soma size and Braak stage in the control group (Fig. S8r). Morphologically, microglial soma appeared round and uniform in size across all groups, with no noticeable changes in the extent of their ramifications (Fig. 4a). Moreover, in non-diabetic controls, Iba1-ir soma size was negatively correlated with both ChAT-ir area and optical density (Fig. S9c, f). A positive correlation between Iba1-ir cell density and ChAT-ir optical density was observed in both controls and T2DM subjects (Fig. S9d), suggesting that microglial activity may be linked to cholinergic neuronal integrity in the NBM.

### The impact of diabetes on astroglia and the glymphatic system

**Fig. 5:**
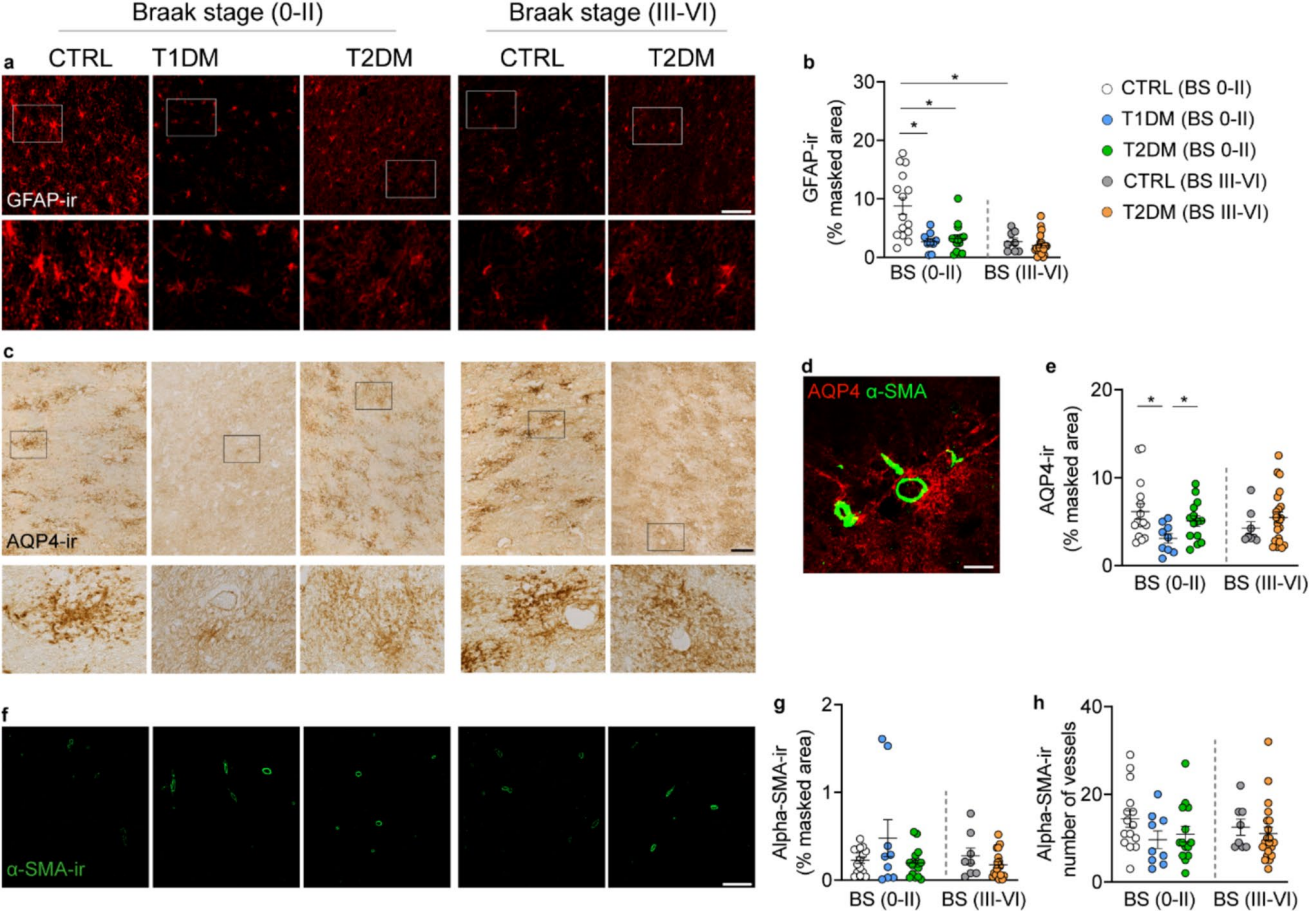
Differential impact of diabetes on astroglia and glymphatic system. **a** Representative images of glial fibrillary acidic protein immunoreactive (GFAP-ir) in the NBM of control, T1DM, and T2DM subjects; high-magnification views of the boxed areas are shown in the lower panels. **b** Quantification of the GFAP-ir masked area (%) in control, T1DM, and T2DM subjects with Braak stage 0–II or III–VI. GFAP-ir is reduced in both T1DM and T2DM subjects with BS 0–II, as well as in controls with BS III–VI. **c** Representative images of aquaporin 4 immunoreactive (AQP4-ir) in the NBM of control, T1DM, and T2DM subjects; high-magnification views of the boxed areas are shown in the lower panels. **d** Illustration of AQP4-ir astrocytes surrounding alpha-SMA-ir vessels that form the peri-vascular glymphatic system. **e** Comparisons of the masked area of AQP4-ir between control, T1DM, and T2DM in BS 0–II or BS III–VI. AQP4-ir is significantly lower in T1DM subjects compared to controls and T2DM with Braak stage 0–II.** f** Representative images of alpha-smooth muscle actin immunoreactive (α-SMA-ir) in the NBM of the control, T1DM, and T2DM subjects. **g, h** Comparisons of the alpha-SMA-ir masked area and the number of stained vessels between control, T1DM, and T2DM with BS 0–II or III–VI. Scale bar: 30 µm in **a,** **d**, 50 µm in **c**, 20 µm in **f**. Data are represented as mean ± SEM. * *p* < 0.05

In addition to microglia, astrocytes also play a significant role in AD pathogenesis [1, 19]. We found both T1DM and T2DM with Braak stage 0–II had lower GFAP-ir astrocytes compared to controls with Braak stage 0–II. Moreover, controls with Braak stage III–VI showed reduced numbers of GFAP-ir astrocytes compared to the controls with Braak stage 0–II. In contrast, no difference was observed between T2DM subjects with Braak stage 0–II and those with Braak stage III–VI (Figs. 5a, b). A subpopulation of astrocytes that express AQP4 is a key component of the brain glymphatic system which is crucial for clearing brain waste, including amyloid-β [27, 42]. Previous studies have reported conflicting results on AQP4 levels in AD brains [55, 68]. Interestingly, we found that AQP4-ir was lower in T1DM compared to controls and T2DM subjects with Braak stage 0–II (Figs. 5c–e). Conversely, AQP4-ir in controls with low or high Braak stages did not show differences, suggesting that AQP4 levels in the NBM are not associated with AD pathology. However, confounder analysis (Fig. S10) revealed a positive correlation between AQP4-ir and ChAT-ir in control subjects (Fig. S10g, h), indicating that astrocytic dysfunction may be associated with cholinergic neuron loss in the NBM.

Morphologically, in controls with Braak stage 0–II, GFAP-ir astrocytes exhibited a typical stellate morphology, characterized by slender, well-branched processes. In contrast, astrocytes in both T1DM and T2DM subjects appeared sparser and displayed thinner, less ramified processes, indicative of a quiescent or less reactive state. AQP4 immunoreactivity showed a generally clustered distribution (Fig. 5c–e) across all groups, with no overt morphological differences. However, a clear reduction in AQP4-labeled area was observed in T1DM subjects. These findings suggest that astrocytes are selectively affected by T1DM-associated neuropathology.

Given the close association between GFAP- and AQP4-expressing astrocytes and the vasculature, we also examined alpha-smooth muscle actin (α-SMA), a marker of capillary pericytes and smooth muscle cells in arterioles and arteries. While a previous study reported increased α-SMA-immunoreactive (α-SMA-ir) vessels in the hypothalamus of T2DM individuals [78], we found no significant differences in α-SMA-ir area across control, T1DM, and T2DM groups at different Braak stages (Figs. 5f–h). However, confounder analysis (Figs. S11 and S12) revealed a positive correlation between GFAP-ir and blood glucose levels in the T1DM group (Fig. S11d), and a negative correlation between α-SMA-ir masked area and glucose levels in the same group (Fig. S12j).

### Confounder analysis

In addition to the aforementioned factors in confounder analyses (Figs. S1–S12), we found a positive correlation between ChAT-ir and PMD in the control group and with CSF pH value in the T1DM group (Figs. S2b, 2c). There was a negative association between CP13-ir and PMD in the control group (Fig. S5B) and a negative correlation of AT8 with fixation time and PMD in the T2DM group (Fig. S6k). Additionally, we observed correlations between AT8 and PMD in controls (Figs. S8b, 8 h, 8n) and CSF pH in T2DM (Figs. S8c, 8i). Moreover, there was a positive correlation between Iba1-ir soma size and Braak stage in control subjects (Fig. S8) and a negative correlation between GFAP-ir masked area and Braak stage in both control and T2DM subjects (Fig. S11), indicating that cognitive decline is associated with neuroinflammatory changes in glial cells in the NBM. Further incidental significance for several parameters is shown in Figures S1–S12. Nevertheless, these correlations did not affect our results because the groups were matched for these factors. Furthermore, Fisher’s z-tests of all positive and negative correlation coefficients showed no significant differences among control, T1DM, and T2DM groups (Supplementary Table 2), indicating statistical equivalence of these correlations across groups.

Regarding the potential effects of anti-diabetic treatments on neuronal and glial markers, we compared all immunostaining outcomes in subjects treated with or without insulin or metformin. No significant differences were observed between these selected treatment groups (Figs. S13 and S14). Additionally, we assessed the influence of ApoE genotype by stratifying subjects accordingly; no significant differences in the main outcomes were found across ApoE subtypes (Fig. S15).

## Discussion

To investigate the association between diabetes and cognitive decline, we studied neurons, glial cells, and vasculature in the NBM using postmortem brain tissues donated by people with T1DM or T2DM, and those without diabetes as matched controls. We found significantly less ChAT-ir in the NBM of people with T1DM, which correlated with reduced Golgi apparatus matrix protein GA130-ir in NBM neurons, as well as diminished Iba1-ir microglia and AQP4-expressing astrocytes in the same region. This discovery highlights a unique neuron-glia dysfunction associated with T1DM, indicating that reduced NBM activity and potentially impaired ACh production may underlie increased cognitive vulnerability in this group.

Given their younger age at death and prior evidence implicating cholinergic deficits in cognitive decline [17], individuals with T1DM may be at elevated risk for developing cognitive impairment as they age, despite the absence of overt clinical symptoms at the time of death. This is consistent with earlier studies showing that cholinergic dysfunction in AD may begin during the preclinical phase—prior to the onset of measurable cognitive symptoms [3, 41, 52]. Furthermore, our data showed that among individuals with Braak stage III–VI, those with T2DM exhibited more p-Tau-ir in the NBM compared to the non-diabetic controls, suggesting that T2DM may exacerbate neuropathological changes associated with AD.

Regarding the T1DM pathology underlying the pronounced cholinergic neuronal dysfunction, one of the most plausible explanations is the dark side of insulin—hypoglycemia. Compared to T2DM, people with T1DM experience hypoglycemia more frequently. It is estimated to have a prevalence of 50% in T1DM compared to 10% in T2DM [48]. Frequent hypoglycemia results in larger glucose variability, characterized by fluctuations between hyperglycemic and hypoglycemic states [62]. The detrimental impact of hypoglycemia on cholinergic neurons includes disruption of ATP production and mitochondrial function [33], as well as impaired biosynthesis of ACh from the glucose metabolite Acetyl-CoA. These changes can collectively compromise cholinergic neuronal function. Conversely, cognitive impairment can worsen poor glycemic control because it heavily relies on self-management of the person with diabetes [30, 40, 60], thus creating a detrimental vicious cycle that further impairs brain function. Our results also show a decreased trend of the ChAT-ir area in controls with Braak stage III–VI, which is similar to what was found in a previous study [18].

Insulin is known for its neuroprotective role [2, 24]. A previous study has suggested that a combination of insulin and other diabetes medications may reduce p-Tau levels [5]. Insulin has thus been explored as a potential therapy for AD [15]. However, our results suggest that lifelong insulin treatment in T1DM does not prevent cholinergic neuronal dysfunction in the NBM. A potential explanation is that insulin supplementation in T1DM may inadequately reach brain cells due to insulin resistance [53, 77]. The blood–brain barrier can limit insulin transport into the CNS during the circumstances of hyperinsulinemia or insulin resistance [32, 57], which could reduce insulin’s neuroprotective effects despite systemic therapy.

Future experimental studies should investigate whether optimizing insulin delivery to the brain—such as through intranasal administration—can enhance its protective effects on cholinergic neurons and tau pathology. Additionally, exploring the interaction between peripheral and CNS insulin resistance in T1DM and their combined impact on neurodegeneration could provide critical insights. Such findings in animal models may guide the development of more effective therapeutic strategies targeting the underlying mechanisms of AD pathology.

Despite more frequent hypoglycemia in T1DM, both persons with T1DM and T2DM commonly experience hyperglycemia, which may explain our findings of lower GFAP-expressing astrocytes in both groups. Previous studies have also reported a loss of GFAP-positive astrocytes in other brain regions of T2DM subjects [23], although the mechanisms underlying hyperglycemia-induced astrocytic dysfunction require further investigation. Hyperglycemia’s adverse effects on blood vessels [21, 49, 61] and its association with increased alpha-SMA in AD [26, 66] were not observed in the NBM, suggesting that vascular dysfunction may not contribute to cholinergic neuronal loss in T1DM. Regarding other pathological changes associated with hyperglycemia, a previous study using streptozotocin-induced T1DM animal models found a lower production of ACh in striatal slices in culture, but did not detect a difference in freshly isolated striata and hippocampal tissues [73].

While T2DM subjects showed comparable ChAT-ir to controls, epidemiological studies link T2DM with cognitive impairments [64]. Indeed, in subjects with Braak stage III–VI, we observed higher expression of CP13-ir, AT8-ir, and PHF1-ir in T2DM subjects, indicating exacerbated neuropathological changes associated with AD progression. Elevated p-Tau levels have also been reported in the CSF of people with T2DM compared to healthy controls [39], consistent with our findings and epidemiological data. Moreover, our previous studies have observed less neurons that express proopiomelanocortin in the infundibular nuclei, fewer oxytocin neurons in the paraventricular nuclei, as well as reduced arginine vasopressin and vasoactive intestinal polypeptide in the suprachiasmatic nuclei [11, 23, 31], suggesting that T2DM does affect neuronal function in various brain regions.

Microglia and the glymphatic system both function as keepers of brain homeostasis [10, 25, 28, 42, 71, 76], yet their roles in supporting cholinergic neuronal function remain unclear. Unlike activated microglia in AD brains [37], we observed a reduction in microglial cells in the NBM of people with T1DM. This reduction does not appear to be caused by hypoglycemia or hyperglycemia, since previous animal studies have shown that microglia become activated in response to hypoglycemia [75]. Moreover, in previous studies of our group, using diabetic *db*/*db* mice (known for uncontrolled hyperglycemia), we also did not observe a decrease in microglial cell density [16]. An alternative explanation could be that reduced cholinergic neuronal activity in the NBM leads to a decreased immune demand compared to normal physiological conditions, resulting in lower microglial activity over time and subsequently fewer microglial cells in the NBM. In contrast to microglia, studies investigating AQP4 levels in AD brains have produced conflicting results [55, 68]. Our finding of lower AQP4 expression in T1DM brains raises questions about whether reduced glymphatic activity is due to frequent hypoglycemia or decreased immune activity resulting from neuronal loss, as hypothesized for microglia alterations.

*Limitations of the study:* Although the tissue collection spans more than 40 years, the number of donors with a clearly documented diagnosis of T1DM was very limited. As a result, the T1DM group consisted of only nine individuals. While our analyses focused on cholinergic dysfunction, these donors may also have had other complications of T1DM, this may introduce heterogeneity that could influence neuropathological outcomes. Moreover, all T1DM donors in this study were classified as Braak stage 0–II, which prevented comparisons with later Braak stages and limited our ability to assess whether T1DM contributes to AD-like pathology as disease severity progresses. Such small and heterogeneous sample sizes may increase variability and constrain the generalizability of our findings. Therefore, these results should be considered preliminary and warrant confirmation in studies with larger, more uniformly characterized cohorts that include donors across the spectrum of Braak stages.

In conclusion, our finding of reduced ChAT-ir neurons in the NBM of people with T1DM, for the first time provides a potential mechanistic link between T1DM and AD. This finding also refers to the “cholinergic hypothesis,” one of the earliest theories of AD pathogenesis [4]. We suggest measuring cholinergic index, i.e., ratio of ChAT/AChE (acetylcholinesterase) in the CSF, as a potential biomarker for diagnosing cognitive dysfunction in people with T1DM. If confirmed, acute testing with an acetylcholinesterase inhibitor may determine whether enhancing cholinergic neurotransmission improves cognitive performance. This may lead to considering the cholinergic index as a biomarker indicating early stages of cognitive decline. Additionally, enhancing cholinergic neurotransmission through supplementation with AChE inhibitors, such as Donepezil, which is used to treat symptoms of Alzheimer’s disease, could be considered as a therapeutic approach to improve cognitive performance in people with T1DM.

## Supplementary Information

Below is the link to the electronic supplementary material.

Supplementary file1 (PDF 5633 KB)
