# Supplementary material for: Neuropathological changes in the nucleus basalis of Meynert in people with type 1 or type 2 diabetes mellitus
Source: Acta Neuropathol. 2025 Sep 29;150(1):35. doi: 10.1007/s00401-025-02942-y (PMC12479689; doi:10.1007/s00401-025-02942-y)
Supplement: Supplementary file 1 — Supplementary file1 (PDF 5633 KB) [file 401_2025_2942_MOESM1_ESM.pdf]

Neuropathological changes in the nucleus basalis of Meynert in people with type 1 or type 2 diabetes mellitus

Wei Jiang, Martin J. Kalsbeek, Felipe Correa-da-Silva, Han Jiao, Andries Kalsbeek, Dick F. Swaab, Sarah E. Siegelaar, Chun-Xia Yi.

Supplementary material

Contents

Supplementary Information for Methods.

Supplementary Table 1: Antibodies information.
Supplementary Table 2: Comparison of correlation coefficients (Fisher's z-test).

Supplementary Figure 1: Neuron density visualized by Nissl staining in the NBM of control, T1DM, and T2DM subjects.
Supplementary Figure 2: Confounder analysis of ChAT-ir in the NBM of control, T1DM, and T2DM subjects.
Supplementary Figure 3: Correlation analysis of CDR and MMSE scores with ChAT-ir in the NBM of control and T2DM with Braak stage III-VI.
Supplementary Figure 4: Confounder analysis of GA-ir in the NBM of control, T1DM, and T2DM subjects.
Supplementary Figure 5: Confounder analysis of CP13-ir in the NBM of control, T1DM, and T2DM subjects.
Supplementary Figure 6: Confounder analysis of AT8-ir in the NBM of control, T1DM, and T2DM subjects.
Supplementary Figure 7: Confounder analysis of PHF-ir in the NBM of control, T1DM, and T2DM subjects.
Supplementary Figure 8: Confounder analysis of Ibal-ir in the NBM of control, T1DM and T2DM subjects.
Supplementary Figure 9: Correlation between Iba1-ir and ChAT-ir in the NBM of control, T1DM, and T2DM subjects.
Supplementary Figure 10: Confounder analysis of AQP4-ir in the NBM of control, T1DM, and T2DM subjects.
Supplementary Figure 11: Confounder analysis of GFAP-ir in the NBM of control, T1DM and T2DM subjects.
Supplementary Figure 12: Confounder analysis of alpha-SMA-ir in the NBM of control, T1DM, and T2DM subjects.
Supplementary Figure 13: Comparative analysis of all markers in T1DM and T2DM Subjects with and without insulin treatment.
Supplementary Figure 14: Comparative analysis of all markers in T1DM and T2DM Subjects with and without Metformin treatment.
Supplementary Figure 15: Comparative analysis of all immuno-stained markers in T1DM and T2DM subjects stratified by ApoE sub-genotype.

Supplementary Information for Methods

Nissl staining
Sections were mounted on glass slides (superfrost+, Thermo Scientific) and dried on a 37°C heating plate. After 48 hours, the sections were deparaffinized in 100% xylene, rehydrated in grading ethanol (100% - 50%) and rinsed in distilled water. Next, the sections were submerged in a 0.5% thionine solution for 5 minutes and rinsed in water. After dehydration in graded ethanol (50%-100%) and xylene, sections were cover slipped using Entellan (Sigma-Aldrich, 107960, dried by air and ready for analysis.

Heat-induced epitope retrieval
Sections were submerged in citrate buffer (pH 6.0) for the ChAT, Iba1, GFAP, alpha-SMA, Aβ and citrate buffer (pH 9.0) for GA130 and Phospho-TDP43. Afterward, sections were heated for 10 minutes by microwave treatment (700 W), and were subsequently cooled down for 30 minutes; for the CP13 and PHF1 antibodies, we performed epitope retrieval in room temperature by submerging the sections in formic acid (pH 2.0) for 10 minutes.

Immunohistochemistry
After overnight primary antibody incubation, sections were rinsed in TBS and incubated for 60 minutes with biotinylated secondary antibody (1:400, Vector Laboratories). Then incubated for 60 minutes with avidin-biotin complex (1:800, Vectastain Elite ABC kit; Vector Laboratories Inc.) and were subsequently rinsed in TBS. Finally, sections were incubated in 0.5mg/ml 3,3ʹ-Diaminobenzidine (Sigma Chemical Co., St. Louis, MO, DAB) in TBS. For ChAT, Iba1, Phospho-TDP43, Aβ and GA staining, 0.2% ammonium nickel sulphate and 0.01% H2O2 (Merck, Darmstadt, Germany) were added (DAB/Ni) (BDH; Brunschwig, Amsterdam, The Netherlands).

Supplementary Table 1   Antibodies information

Primary antibody	Source	Host	Catalog number	Specificity (PMID)	Dilution
Iba1	Synaptic Systems	Rabbit	234003	32814716	1:400
GA130	Netherlands Institute for Brain Research	Rabbit	α-HG-130	11222994	1:4000
GFAP	DAKO	Rabbit	Z0334	32422642	1:1000
AQP4	Atlas antibodies	Rabbit	HPA014784	27893874	1:1000
Alpha-SMA	Sigma Aldrich	Mouse	A5228	24024123	1:1000
ChAT	Sigma Aldrich	Goat	AB1440P	26792551	1:500
AT8	ThermoFisher	Mouse	MN1020	26792551	1:400
CP13	Gift from Fred van Leeuwen	Mouse	Peter Davies (1)	24788298	1:400
PHF1	Gift from Fred van Leeuwen	Mouse	Peter Davies (1)	24788298	1:400
Amyloid-beta	Abcam	Mouse	AB126649	10707298	1:10000
Phospho-TDP43	Proteintech	Rabbit	80007-1-RR	11249427	1:10000

Reference: (1): Petry FR, Pelletier J, Bretteville A, Morin F, Calon F, Hebert SS, et al. Specificity of anti-tau antibodies when analyzing mice models of Alzheimer's disease: problems and solutions. PLoS One. 2014;9(5):e9425

Supplementary Table 2 Comparison of correlation coefficients (Fisher's z-test)

Correlation markers	Numbers of Individual			r value			P value		
	CTRL	T1DM	T2DM	CTRL	T1DM	T2DM	P[1]	P [2]	P[3]
PMD, CP13-ir area	23	7	35	-0.52	0.27	-0.31	0.12	0.36	0.25
PMD, AT8-ir area	23	7	35	-0.10	0.15	-0.26	0.56	0.52	0.33
PMD, Iba1-ir soma number	23	7	35	0.47	-0.64	-0.02	0.06	0.09	0.18
PMD, Ibal % masked area	23	7	35	0.45	-0.64	-0.04	0.07	0.13	0.16
PMD, Iba1 soma size	23	7	35	-0.48	-0.43	-0.01	1.00	0.11	0.19
PMD, AQP4 % masker area	23	7	35	0.07	0.49	0.42	0.41	0.25	0.77
PMD, ChAT-ir area	23	7	35	0.47	-0.64	-0.06	0.06	0.12	0.14
Age, PHF1-ir O.D.	23	9	36	0.54	0.03	0.23	0.29	0.36	0.57
Age, PHF1-ir area	23	9	36	0.54	-0.22	0.03	0.15	0.09	0.57
Age, AT8-ir	23	9	36	0.46	-0.31	0.15	0.23	0.44	0.29
Age, CP13-ir area	23	9	36	0.43	0.12	0.08	0.87	0.27	0.94
Age, GFAP % masked area	23	9	35	-0.14	-0.73	-0.01	0.18	0.65	0.12
pH CSF, Ibal-ir soma number	18	6	28	0.00	0.71	0.42	0.48	0.34	0.47
pH CSF pH, Ibal % masked area	18	6	28	0.02	0.71	0.40	0.31	0.48	0.45
Fixation time, AQP4 % masked area	23	9	35	0.50	0.33	0.17	1.00	0.56	0.70
Fixation time, AT8-ir area	23	9	35	0.06	-0.04	0.05	0.94	0.94	0.94
Fixation time, GFAP % masked area	23	9	34	0.11	-0.75	-0.09	0.06	0.48	0.10

[1] Control VS T1DM; [2] Control VS T2DM; [3] T1DM VS T2DM; PMD: Postmortem Delay.

Supplementary Figures and Legends

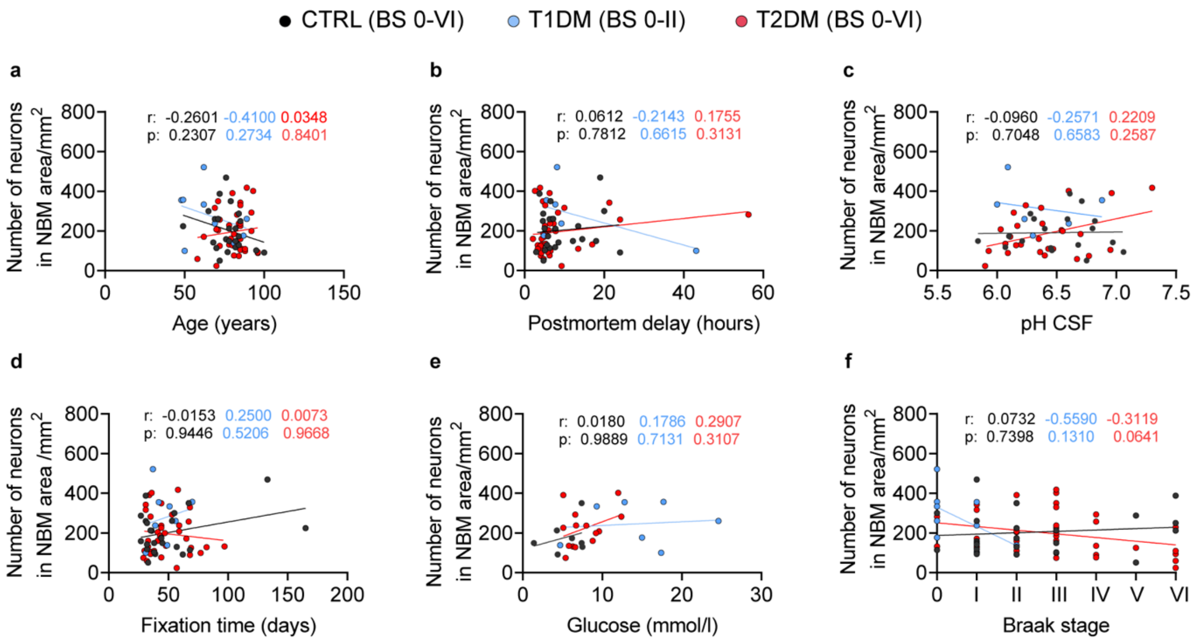

Supplementary Fig. 1: a - f Confounder analysis of the number of thionine-stained neurons in the nucleus basalis of Meynert (NBM) of control (CTRL) and T2DM subjects with Braak stage 0-VI, and T1DM with Braak stage 0-II. No significant correlation was found between thionine-stained cells with these potential confounders.

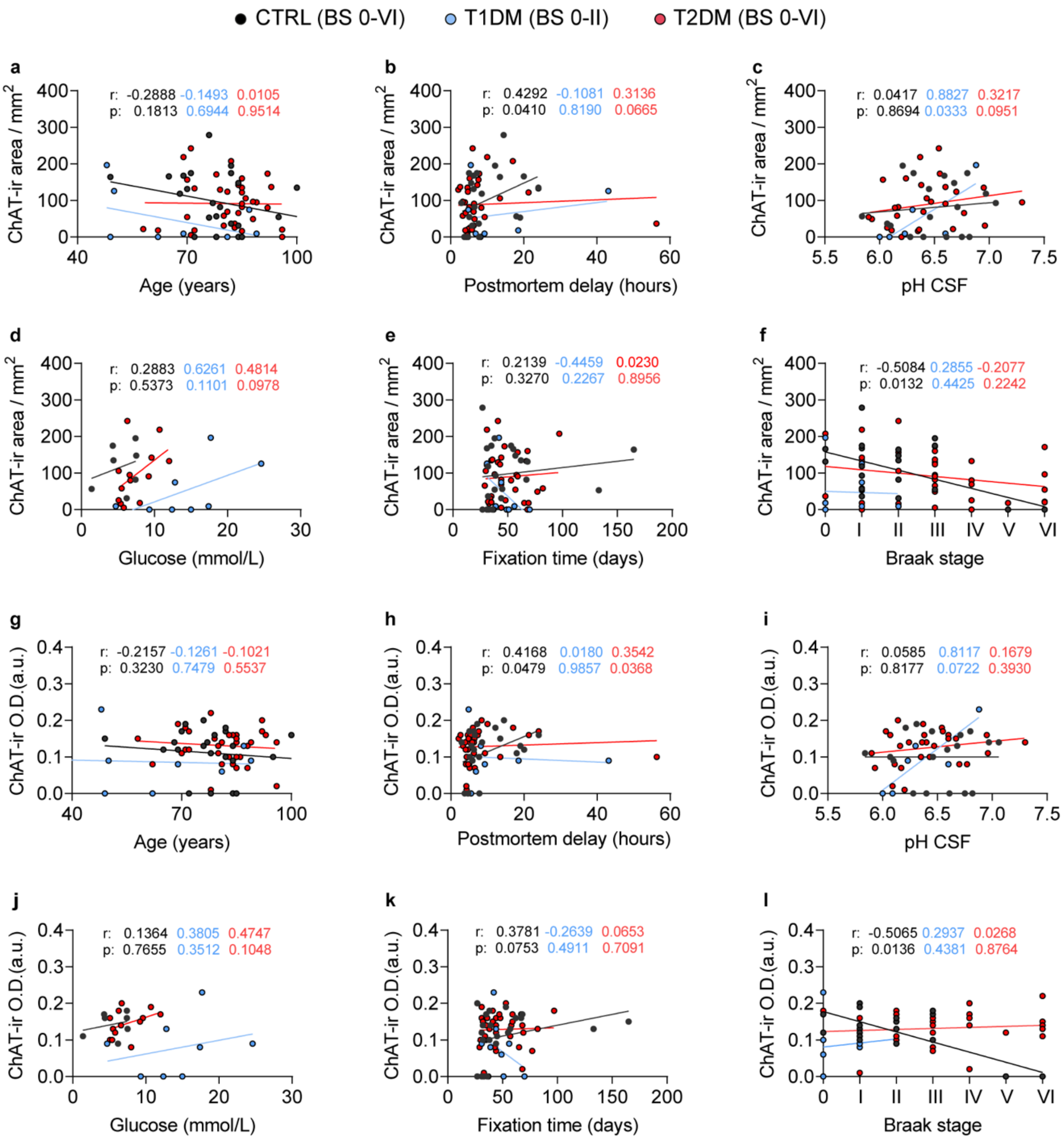

Supplementary Fig. 2: **a - l** Confounder analysis of choline acetyltransferase immunoreactive (ChAT-ir) areas and optical density (in arbitrary unit, O.D. (a.u.)) in the nucleus basalis of Meynert (NBM) of control (CTRL) and T2DM subjects with Braak stage 0-VI, and T1DM with Braak stage 0-II.

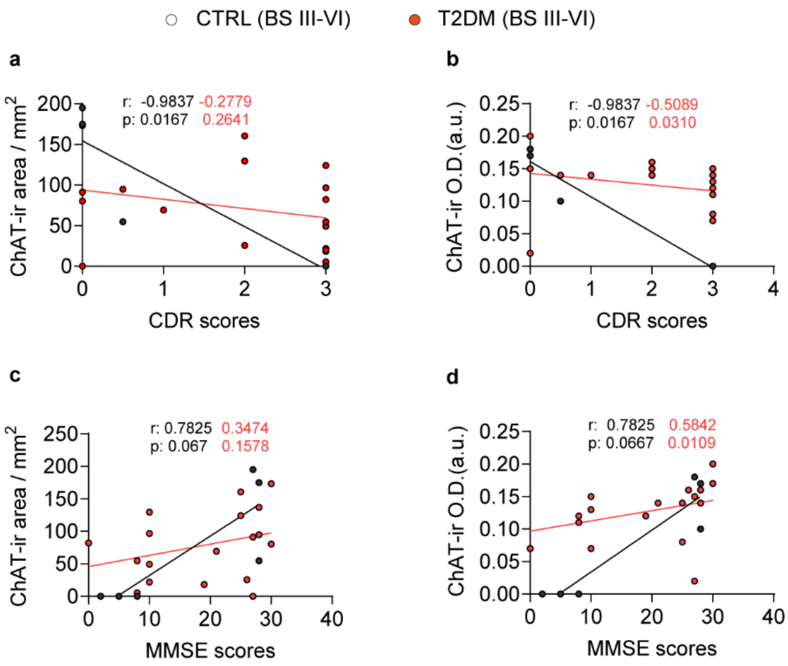

Supplementary Fig. 3a - d Correlation analysis of Clinical Dementia Rating (CDR) and Mini-Mental State Examination (MMSE) scores with choline acetyltransferase-immunoreactive (ChAT-ir) area and optical density (O.D., arbitrary units, a.u.) in the nucleus basalis of Meynert (NBM) of control (CTRL) and T2DM subjects with Braak stage III-VI.

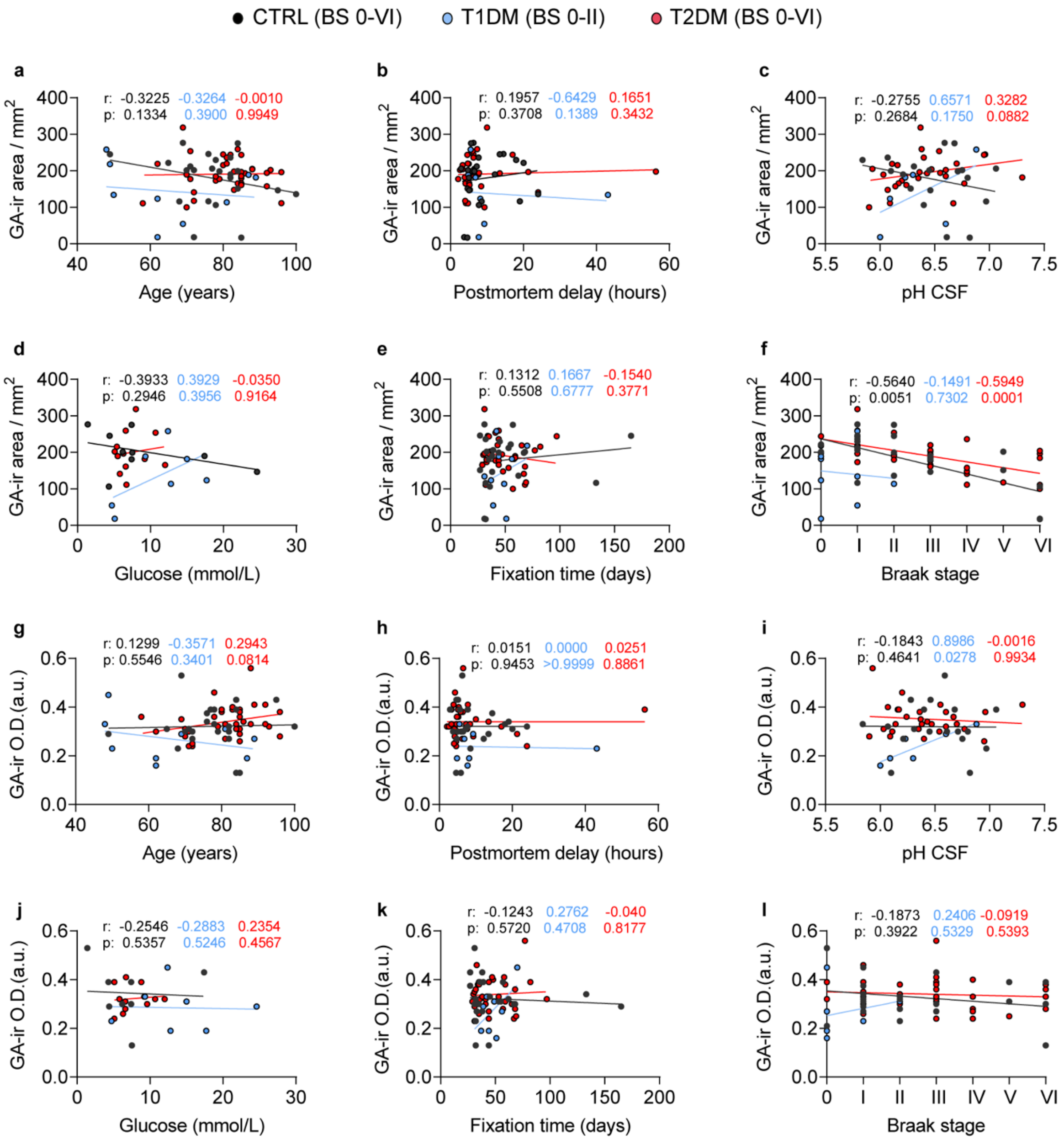

Supplementary Fig. 4 a - l Confounder analysis of Golgi matrix protein GA130 immunoreactive (GA-ir) areas and optical density (in arbitrary unit, O.D. (a.u.)) in the nucleus basalis of Meynert (NBM) of control (CTRL) and T2DM subjects with Braak stage 0-VI, and T1DM with Braak stage 0-II.

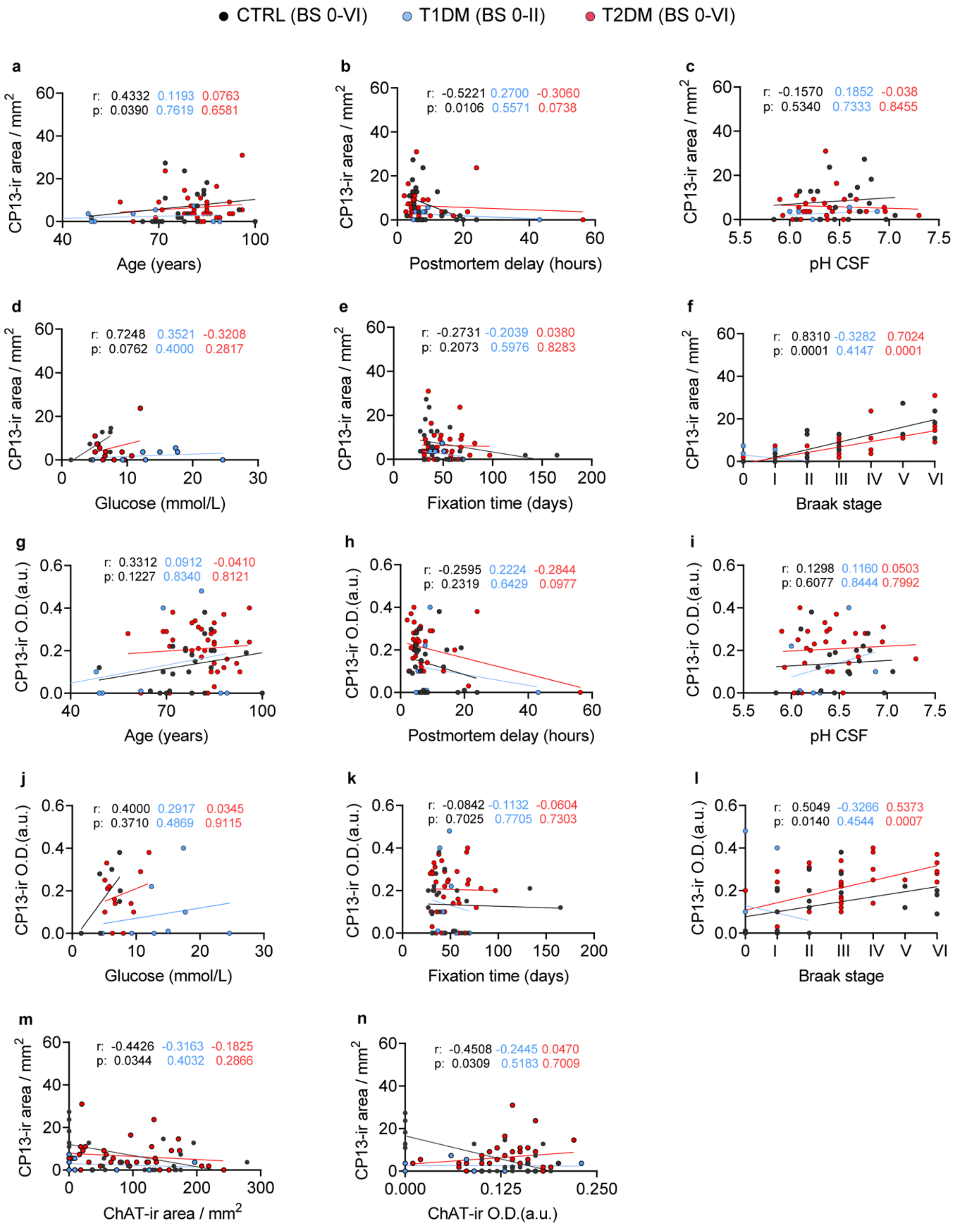

Supplementary Fig. 5 a - l Confounder analysis of p-Tau CP13 immunoreactive (CP13-ir) areas and optical density (in arbitrary unit, O.D. (a.u.)) in the nucleus basalis of Meynert (NBM) of control (CTRL) and T2DM subjects with Braak stage 0-VI, and T1DM with Braak stage 0-II. **m, n** Correlation between the CP13-ir areas and the choline acetyltransferase immunoreactive (ChAT-ir) areas or O.D.

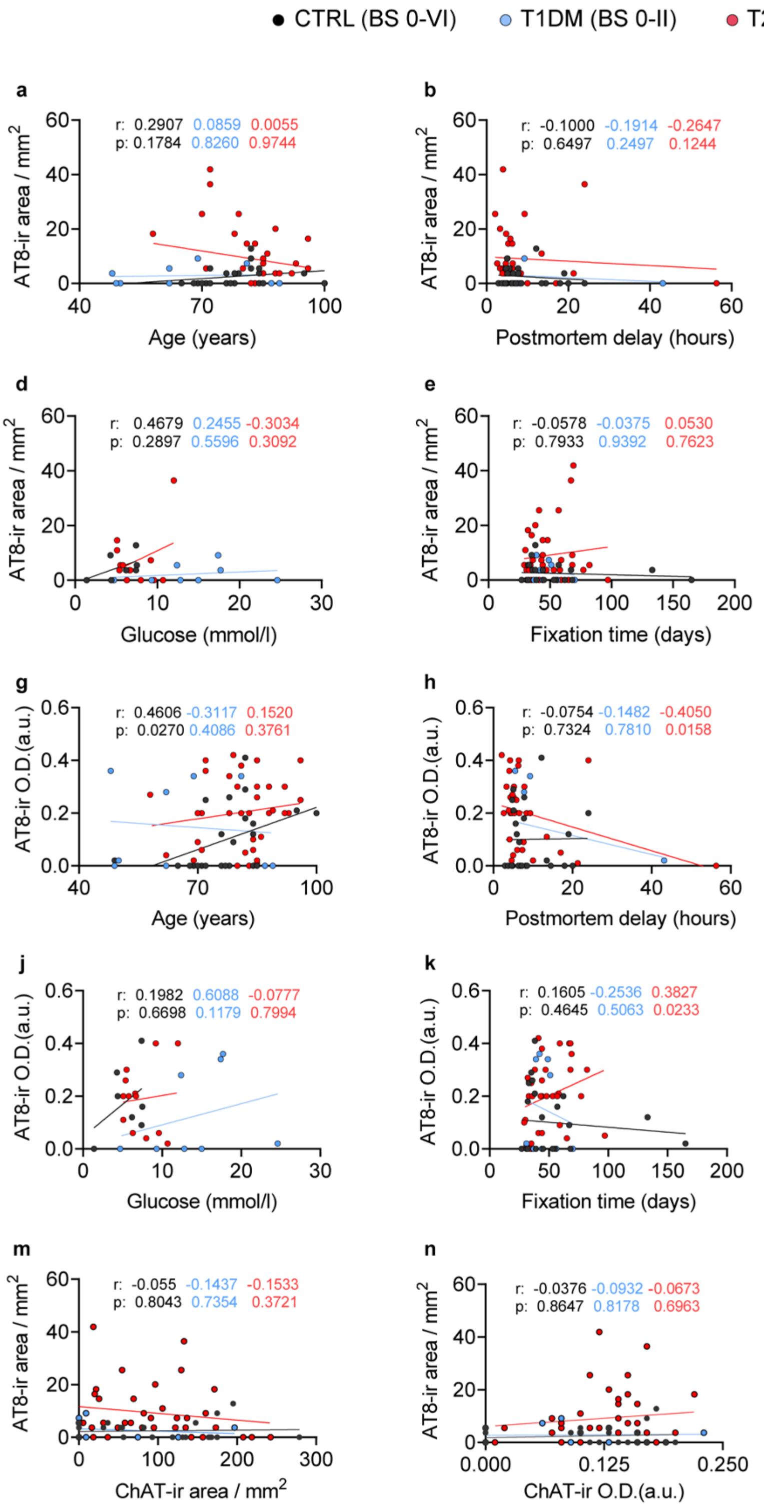

Supplementary Fig. 6 a - l Confounder analysis of p-Tau AT8 immunoreactive (AT8-ir) areas and optical density (in arbitrary unit, O.D. (a.u.)) in the nucleus basalis of Meynert (NBM) of control (CTRL) and T2DM subjects with Braak stage 0-VI, and T1DM with Braak stage 0-II. **m, n** Correlation between the AT8-ir areas and the choline acetyltransferase immunoreactive (ChAT-ir) areas or O.D.

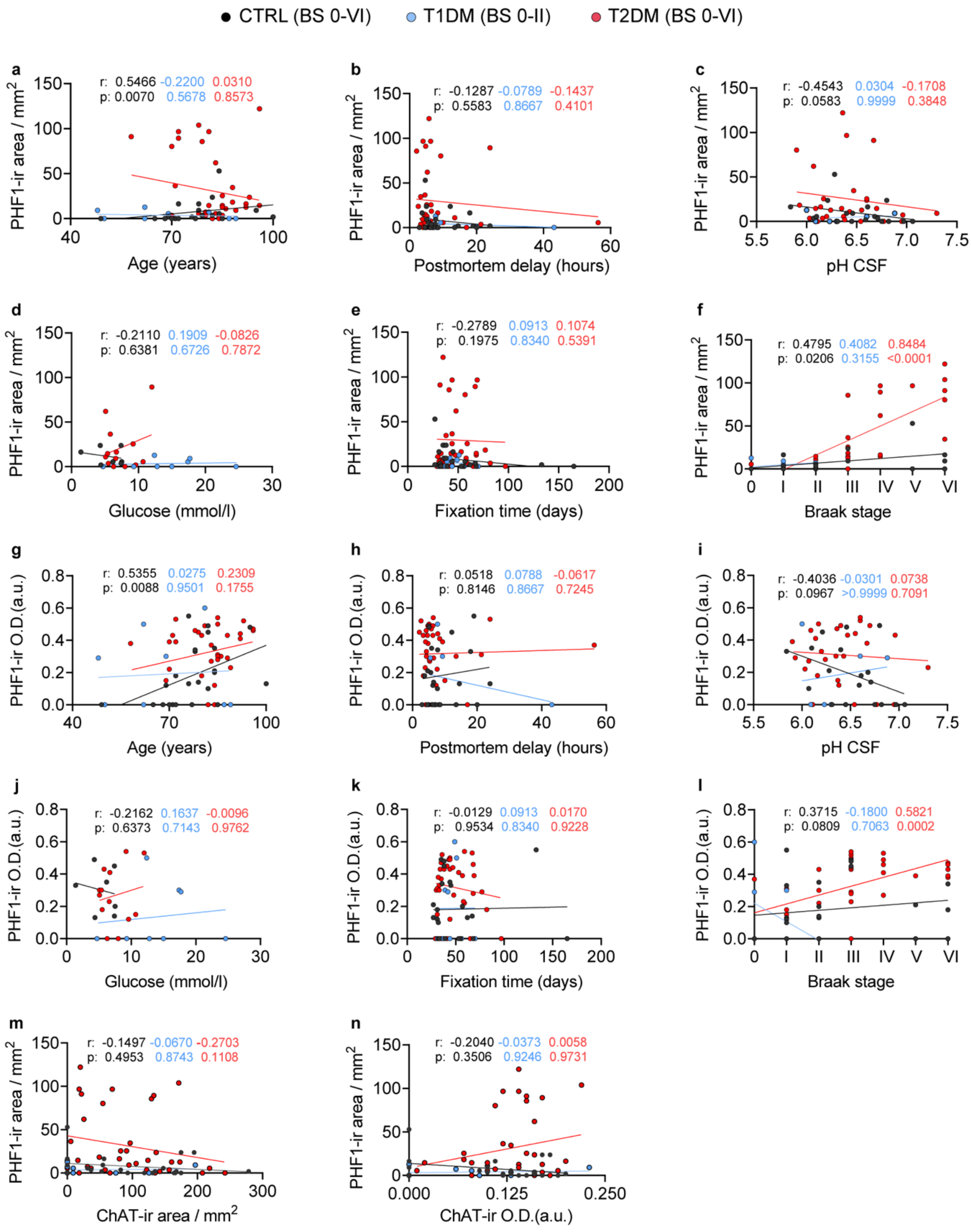

Supplementary Fig. 7 a - l Confounder analysis of p-Tau PHF1 immunoreactive (PHF1-ir) areas and optical density (in arbitrary unit, O.D. (a.u.)) in the nucleus basalis of Meynert (NBM) of control (CTRL) and T2DM subjects with Braak stage 0-VI, and T1DM with Braak stage 0-II. **m, n** Correlation between the PHF1-ir areas and the choline acetyltransferase immunoreactive (ChAT-ir) areas or O.D.

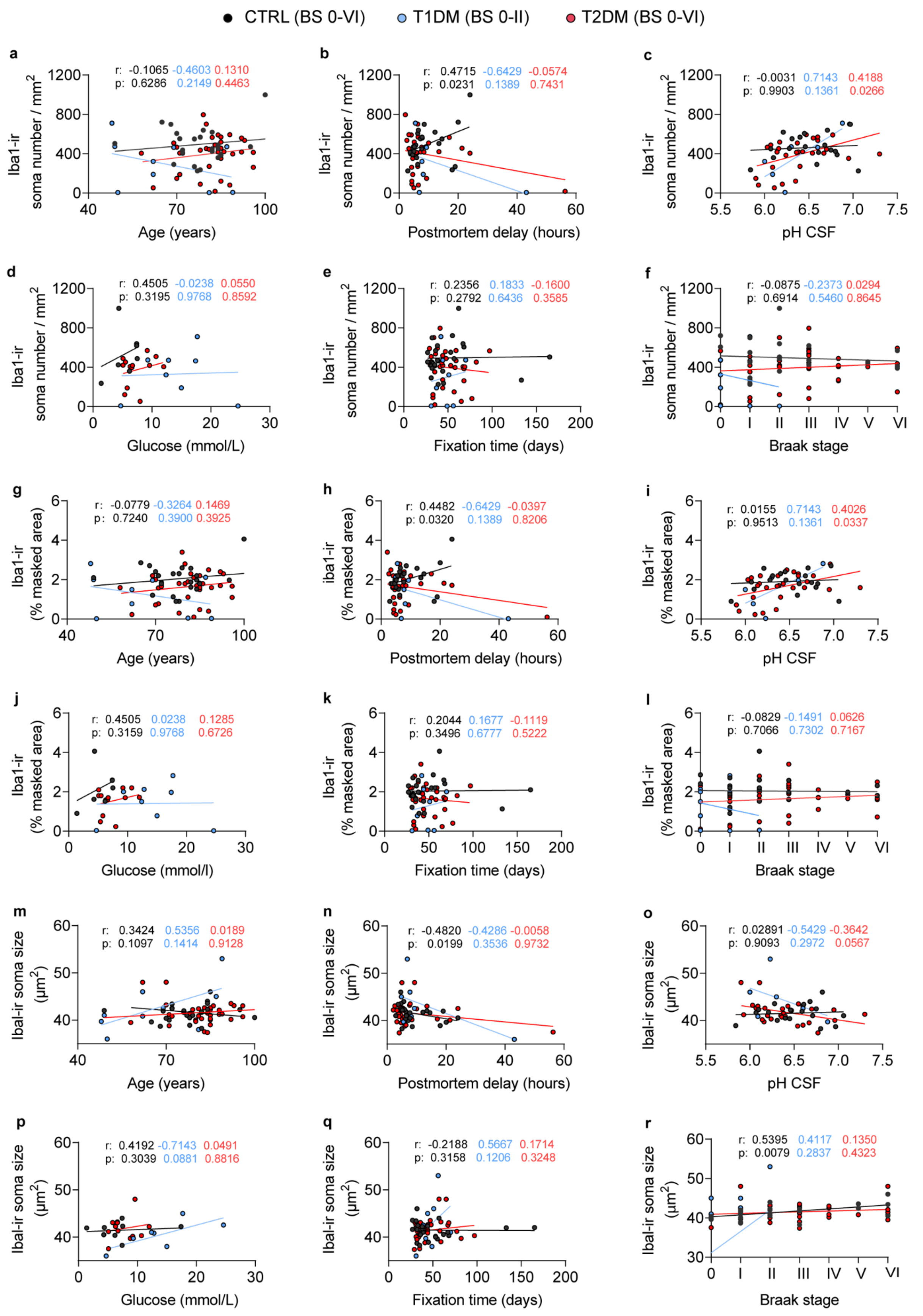

Supplementary Fig. 8 a - r Confounder analysis of ionized calcium binding adaptor molecule 1 immunoreactive (Iba1-ir) soma density, masked area (%) and soma size in the nucleus basalis of Meynert (NBM) of control (CTRL) and T2DM subjects with Braak stage 0-VI, and T1DM with Braak stage 0-II.

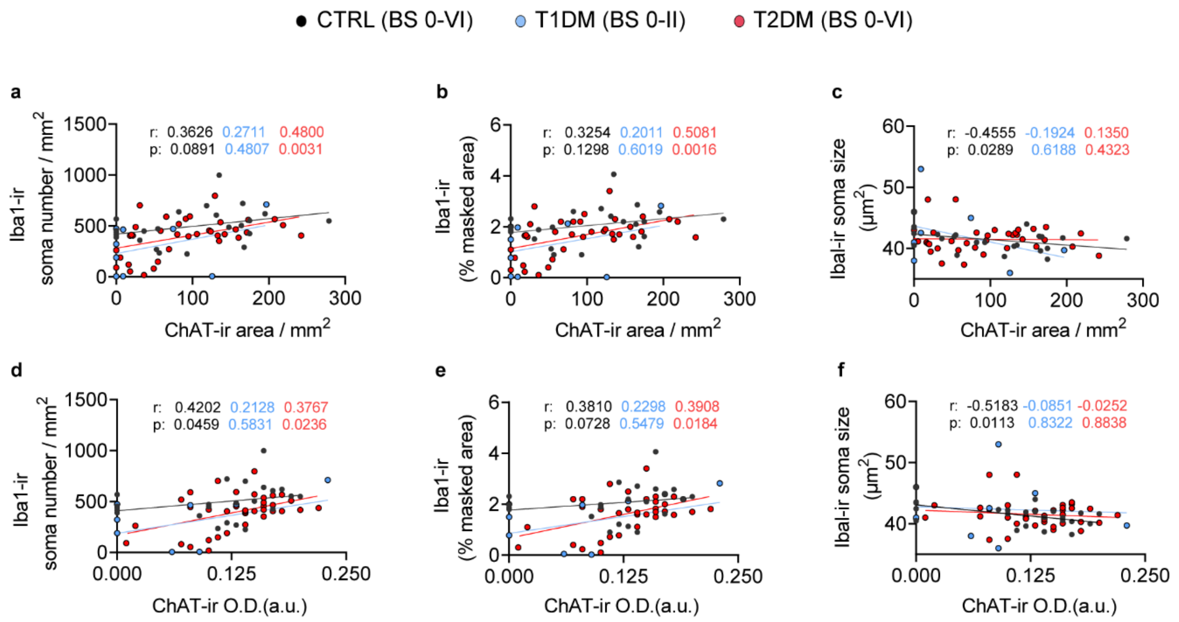

Supplementary Fig. 9 a - f Correlation analysis between the ionized calcium binding adaptor molecule 1 immunoreactive (Iba1-ir) soma density, masked area (%) and soma size with the ChAT-ir areas and optical density (in arbitrary unit, O.D. (a.u.)) in the nucleus basalis of Meynert (NBM) of control (CTRL) and T2DM subjects with Braak stage 0-VI, and T1DM with Braak stage 0-II.

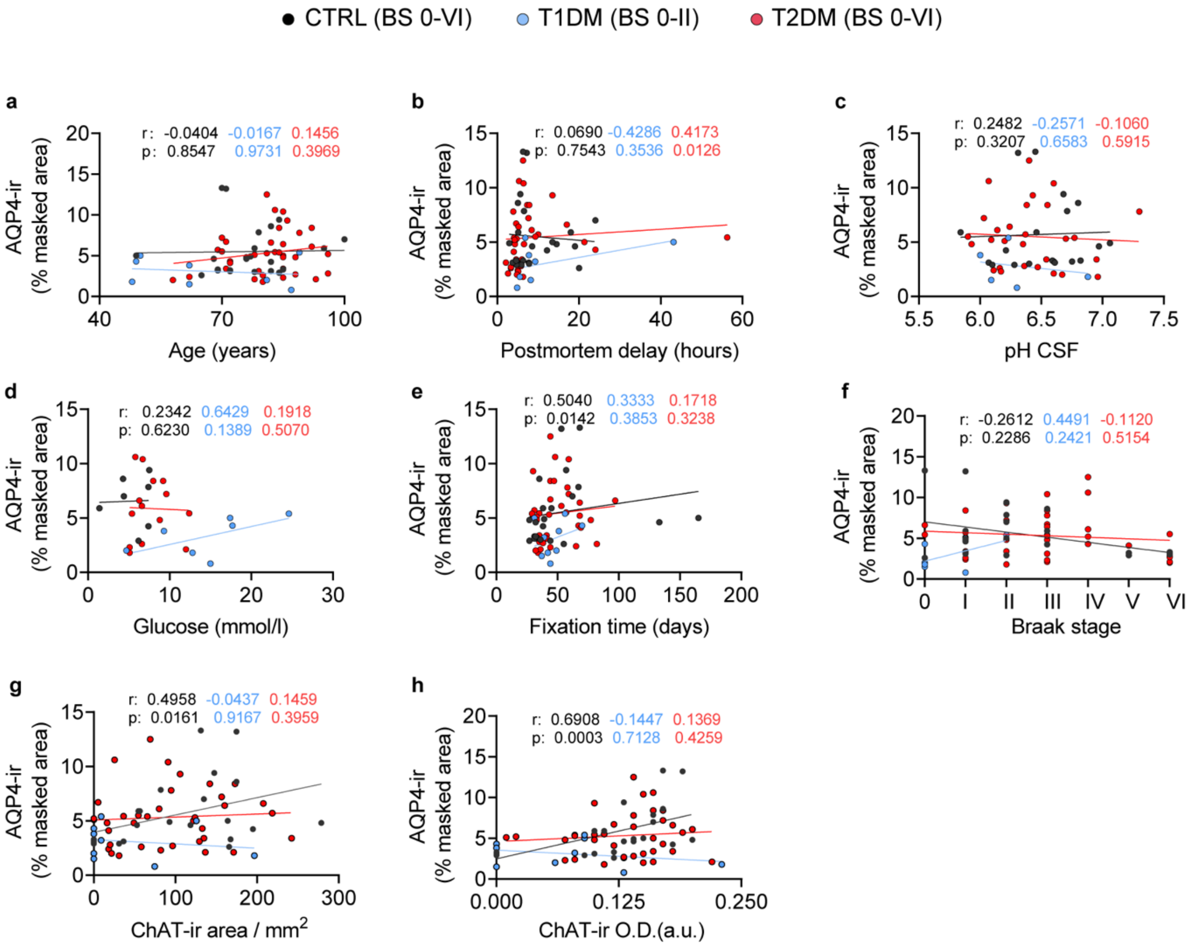

Supplementary Fig. 10 a - f Confounder analysis of aquaporin 4 immunoreactive (AQP4-ir) masked area (%) in the nucleus basalis of Meynert (NBM) of control (CTRL) and T2DM subjects with Braak stage 0-VI, and T1DM with Braak stage 0-II. **g, h** Correlation between the AQP4-ir masked area (%) and the choline acetyltransferase immunoreactive (ChAT-ir) areas and optical density (in arbitrary unit, O.D. (a.u.)).

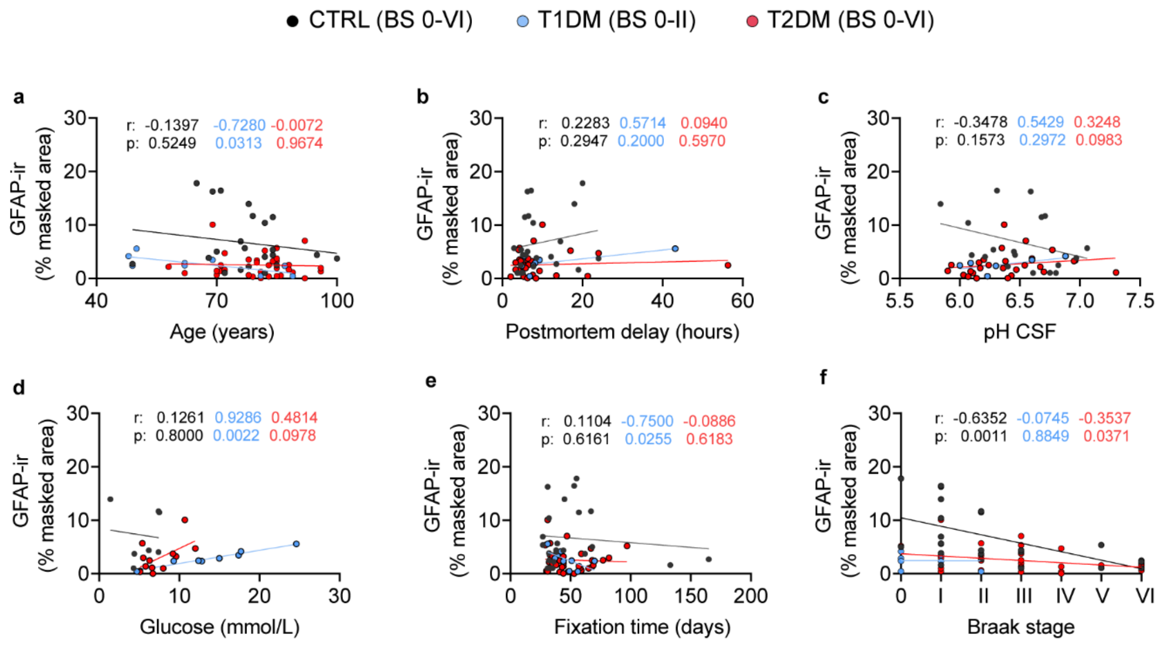

Supplementary Fig. 11 a - f Confounder analysis of glial fibrillary acidic protein immunoreactive (GFAP-ir) masked area (%) in the nucleus basalis of Meynert (NBM) of control (CTRL) and T2DM subjects with Braak stage 0-VI, and T1DM with Braak stage 0-II.

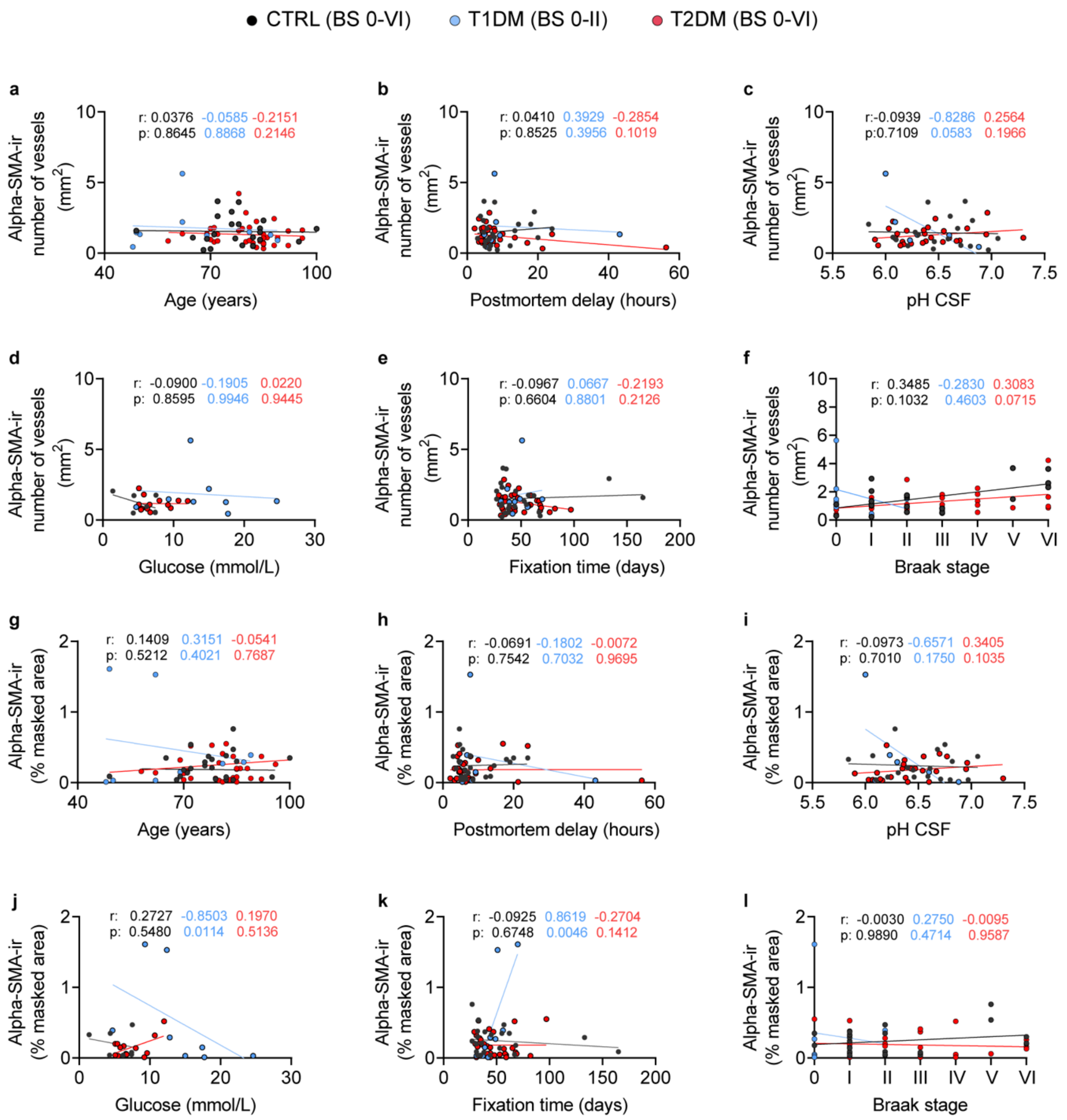

Supplementary Fig. 12 a - l Confounder analysis of alpha-smooth muscle actin immunoreactive (alpha-SMA-ir) vessel density and masked area (%) in the nucleus basalis of Meynert (NBM) of control (CTRL) and T2DM subjects with Braak stage 0-VI, and T1DM with Braak stage 0-II.

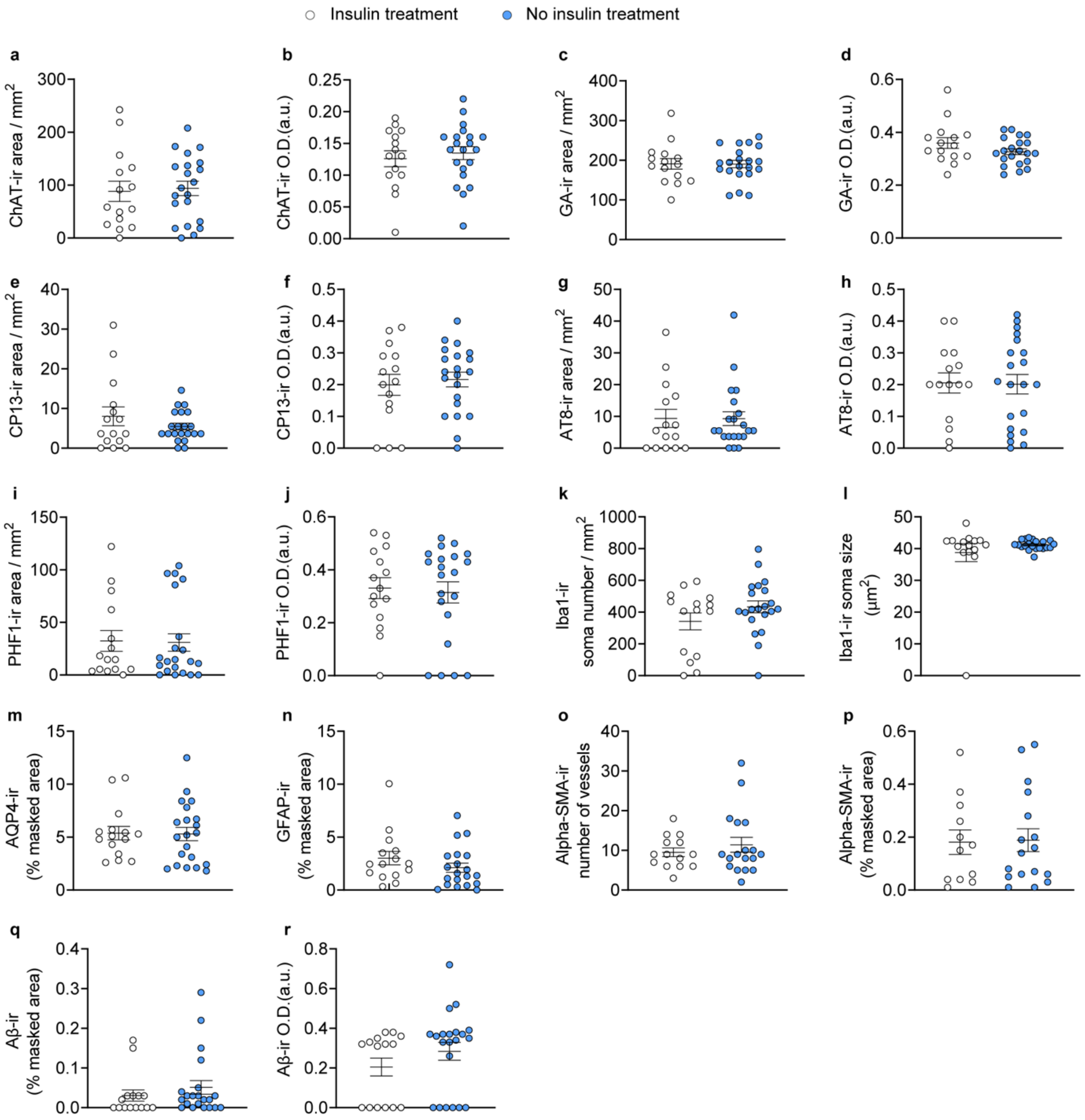

Supplementary Fig. 13 a-r Comparative analysis of all immuno-stained markers in T1DM and T2DM subjects, with or without insulin treatment.

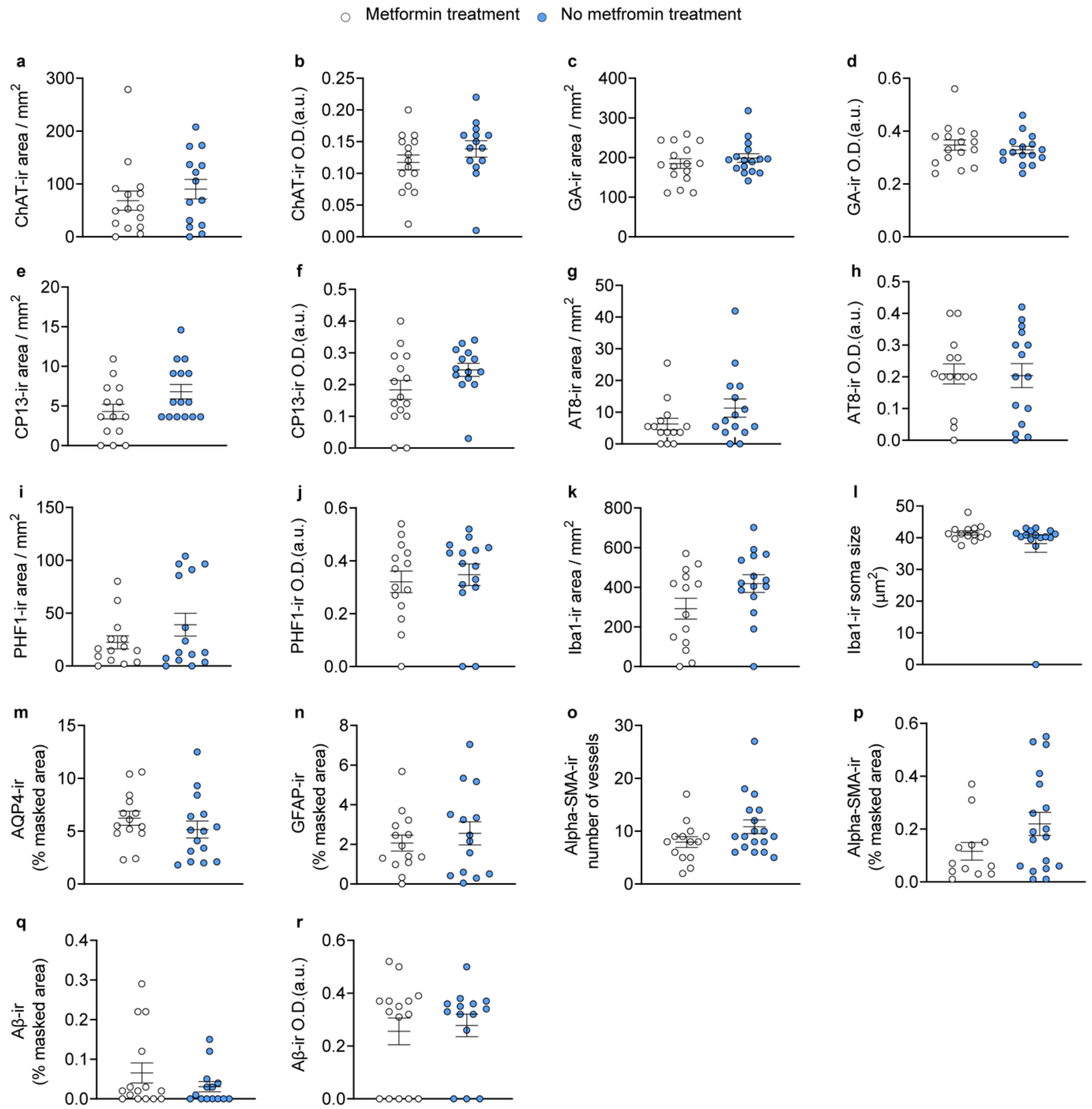

Supplementary Fig. 14 a-r Comparative analysis of all immuno-stained markers in T1DM and T2DM subjects, with or without metformin treatment.

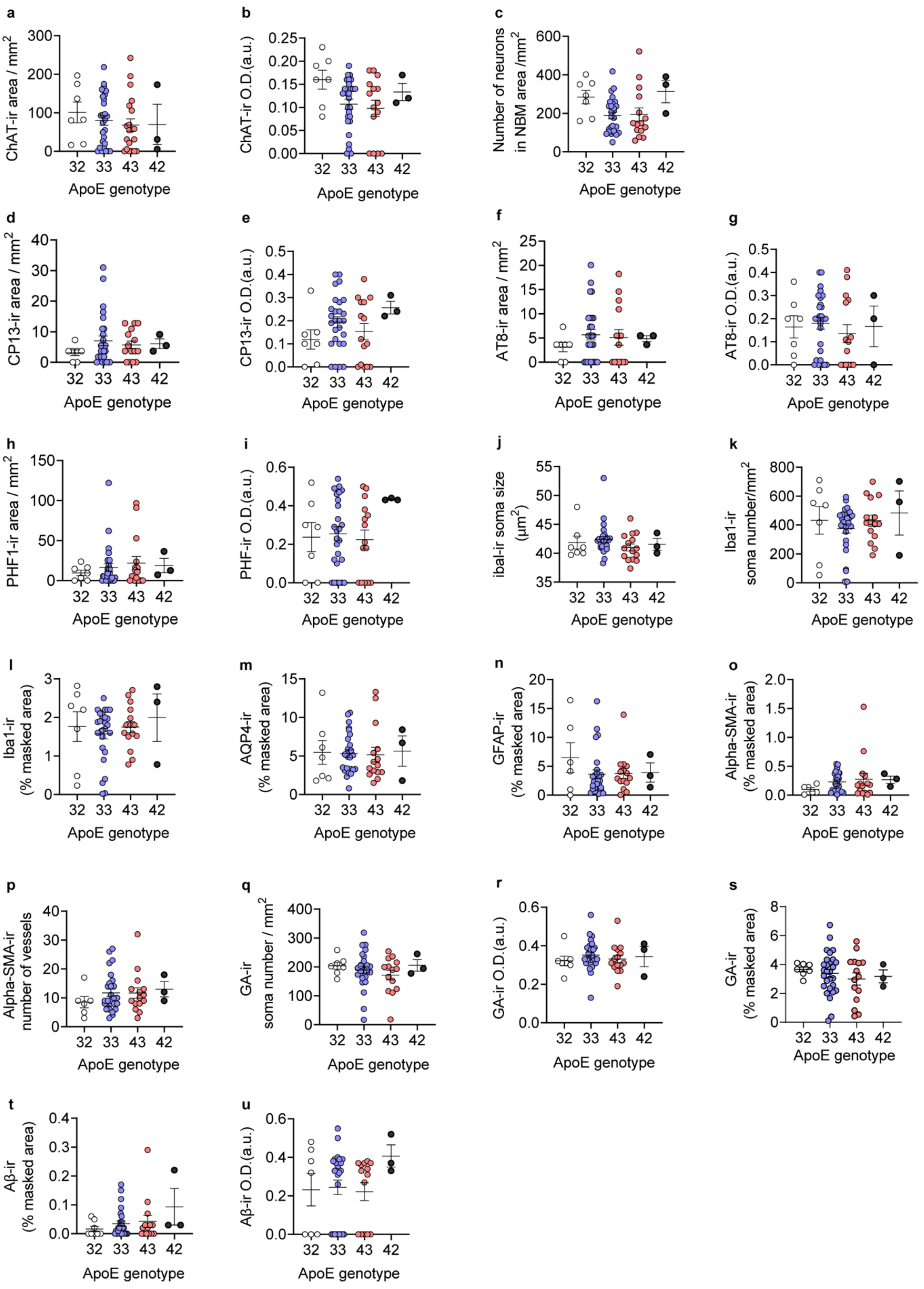

Supplementary Fig. 15 a-u Comparative analysis of all immuno-stained markers in T1DM and T2DM subjects stratified by ApoE sub-genotype.
